# Integrative molecular and clinical profiling of acral melanoma links focal amplification of 22q11.21 to metastasis

**DOI:** 10.1038/s41467-022-28566-4

**Published:** 2022-02-23

**Authors:** Farshad Farshidfar, Kahn Rhrissorrakrai, Chaya Levovitz, Cong Peng, James Knight, Antonella Bacchiocchi, Juan Su, Mingzhu Yin, Mario Sznol, Stephan Ariyan, James Clune, Kelly Olino, Laxmi Parida, Joerg Nikolaus, Meiling Zhang, Shuang Zhao, Yan Wang, Gang Huang, Miaojian Wan, Xianan Li, Jian Cao, Qin Yan, Xiang Chen, Aaron M. Newman, Ruth Halaban

**Affiliations:** 1 Institute for Stem Cell Biology and Regenerative Medicine, Stanford University, Stanford, CA USA; 2 Department of Biomedical Data Science, Stanford University, Stanford, CA USA; 3 IBM Research, Yorktown Heights, NY USA; 4 Xiangya Hospital, Central South University, Changsha, China; 5 Yale Center for Genome Analysis, Yale University, New Haven, CT 06520 USA; 6 Department of Dermatology, Yale University School of Medicine, New Haven, CT USA; 7 Department of Pathology, Yale University School of Medicine, New Haven, CT USA; 8 Department of Internal Medicine, Section of Medical Oncology, Yale University School of Medicine, New Haven, CT USA; 9 Department of Surgery, Yale University School of Medicine, New Haven, CT USA; 10 Department of Molecular and Cellular Physiology, Yale University School of Medicine, New Haven, CT USA; 11 Department of Dermatologic Surgery Institute of Dermatology, Chinese Academy of Medical Sciences & Peking Union Medical College, Nanjing, China; 12 Department of Bone and Soft Tissue oncology, Hunan Cancer Hospital, Affiliated Tumor Hospital of Xiangya Medical School of Central South University, Changsha, Hunan China; 13 Department of Dermatology, The Third Affiliated Hospital, Sun Yat-sen University, Guangzhou, China

**Keywords:** Melanoma, Tumour biomarkers, Tumour heterogeneity, Cancer genomics

## Abstract

Acral melanoma, the most common melanoma subtype among non-White individuals, is associated with poor prognosis. However, its key molecular drivers remain obscure. Here, we perform integrative genomic and clinical profiling of acral melanomas from 104 patients ﻿treated in North America (*n* = 37) or China (*n* = 67). We find that recurrent, late-arising focal amplifications of cytoband 22q11.21 are a leading determinant of inferior survival, strongly associated with metastasis, and linked to downregulation of immunomodulatory genes associated with response to immune checkpoint blockade. Unexpectedly, *LZTR1* – a known tumor suppressor in other cancers – is a key candidate oncogene in this cytoband. Silencing of *LZTR1* in melanoma cell lines causes apoptotic cell death independent of major hotspot mutations or melanoma subtypes. Conversely, overexpression of *LZTR1* in normal human melanocytes initiates processes associated with metastasis, including anchorage-independent growth, formation of spheroids, and an increase in MAPK and SRC activities. Our results provide insights into the etiology of acral melanoma and implicate *LZTR1* as a key tumor promoter and therapeutic target.

## Abstract (web summary)

Despite acral melanoma being the most common melanoma subtype in non-White individuals, its molecular drivers remain unknown. Here, the authors integrate genomic and clinical data from 104 patients and identify late-arising focal amplifications of chr22q11.21 and LZTR1 as a key tumour promoter in this region.

## Introduction

Over the last two decades, a tremendous effort has been made to understand the genomic basis of melanoma. Collectively, these analyses have shown that sun-exposed melanomas harbor a large number of somatic mutations, including single nucleotide variants (SNVs) and genomic rearrangements, associated with ultraviolet (UV) radiation^1–6^. In contrast, acral melanomas, originating from sun-shielded skin such as palms and soles, display a lower SNV mutational burden, a higher rate of structural alteration, and poorer survival outcomes^2,4,7–17^. *BRAF* and *NRAS* are the most frequently affected oncogenes in acral melanomas but at a lower frequency compared to sun-exposed melanomas, whereas *KIT* mutations are more common in acral melanomas^14,18–23^. Copy number variation (CNV) is a well-established feature of acral melanomas, contributing to aberrant regulation of several pathways affecting cell proliferation and gene expression. These include amplification of *CDK4*, *CCND1*, *MAPK1*, and *NOTCH2;* loss of *CDKN2A* (p16^INK4^) and *NF1*; inactivation of *TP53*; modifications of chromatin regulators (e.g., *HDAC* amplification and loss of *ARID1A* and *ARID1B*); and alterations in *TERT*^11,13,14,20,23–25^. Despite these findings, attempts to treat acral melanoma with targeted inhibitors, such as CDK4/6 inhibitors, have failed^12^.

While most genomic studies of acral melanoma have been limited to relatively small clinical cohorts^1,9,13,26,27^, a recent whole-genome analysis of 87 patients—90% of which were of European ancestry—further confirmed the importance of structural rearrangements and copy number aberrations in this disease^15^. Given the predominance of acral melanoma in non-White populations^28,29^ and the lack of effective targeted treatment options, large-scale genomic surveys of acral melanomas from diverse populations are needed.

Here, we apply whole exome (tumor/normal) and RNA sequencing to characterize acral melanomas from 104 patients treated in the United States (*n* = 37) or China (*n* = 67), most of whom had long-term follow-up data available. Through comparative genomic analysis with 157 sun-exposed melanomas, we identify molecular features of acral melanoma; generate a prognostic map linking highly recurrent somatic aberrations in acral melanoma to the risk of death; and find that late-arising focal amplifications in cytoband 22q11.21 are associated with lymph node involvement and distant metastasis. Within 22q11.21, we identify *LZTR1*—a known tumor suppressor in other cancers—as a key candidate driver of metastasis. Our findings reveal molecular insights of acral melanoma pathogenesis and designate LZTR1 as a therapeutic target.

## Results

### Genomic characteristics of acral and sun-exposed melanoma

To characterize the genomic landscape of acral melanoma across diverse patient populations, we analyzed 104 tumors, including 97 by whole-exome sequencing (WES), from patients treated in North America (Yale cohort) or China (CSU cohort) (Table 1). Both cohorts spanned all disease stages, included long-term follow-up, and encompassed patients with diverse ancestry, including White (*n* = 31; Yale) and Asian populations (*n* = 67; CSU) (Supplementary Fig. 1a and Supplementary Data 1). We also applied WES to profile 134 sun-exposed melanoma specimens from patients with stage I through IV disease (Table 1). Notably, sun-exposed melanoma patients showed a longer survival time than acral melanoma patients, consistent with previous studies^16,17^ (Supplementary Fig. 1b). Peripheral blood leukocytes were analyzed as germline controls and whole-transcriptome sequencing (RNA-seq) was applied to 105 tumors, including 38 acral and 37 sun-exposed melanomas with matched WES data (Table 1 and Supplementary Data 1). Tumor purities, clinical follow-up, and median survival times were comparable between acral cohorts, supporting their combined assessment (Supplementary Fig. 1c, d and Supplementary Data 1).

**Table 1:** Patient characteristics and sequencing data.

Characteristics	Acral (*n* = 104)	Sun-exposed (*n* = 157)
Age (years)		
Median (range)	62 (29–89)	66 (20–94)
Sex, *n* (%)		
Female	42 (40)	61 (39)
Male	62 (60)	96 (61)
Stage at tumor resection, *n* (%)		
0	1 (1)	0 (0)
1	10 (10)	7 (4)
2	39 (38)	22 (14)
3	28 (27)	7 (4)
4	26 (25)	121 (77)
Tumor sample site, *n* (%)		
Primary	81 (78)	41 (26)
Metastatic	23 (22)	116 (74)
WES, *n* (%)		
Yale University	32 (33)	134 (100)
Central South University	65 (67)	0 (0)
Bulk RNA-seq, total *n* (*n* with WES)		
Yale University	22 (17)	60 (37)
Central South University	23 (21)	0

Of 104 tumor specimens from patients with acral melanoma, 97 were profiled by WES, 7 were profiled by bulk RNA-seq and not WES, and 45 were profiled by both. Of 157 tumor specimens from patients with sun-exposed melanoma, 134 were profiled by WES, 23 were profiled by bulk RNA-seq and not WES, and 37 were profiled by both.

**Fig. 1:**
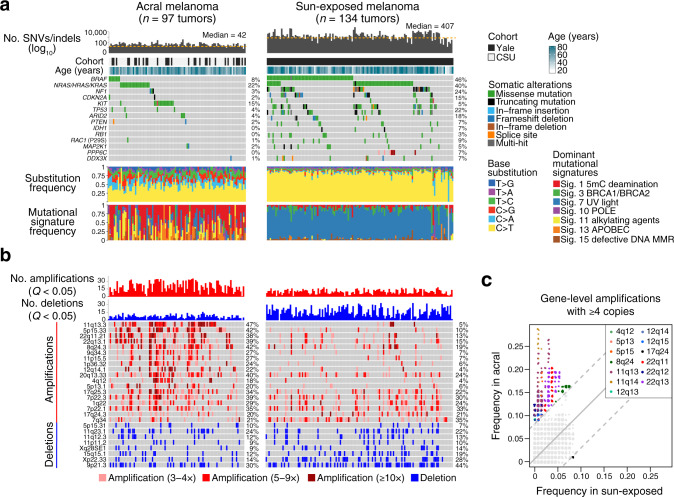
Landscape of somatic alterations in acral and sun-exposed melanomas. **a**, **b** Genomic and clinical characterization of acral and sun-exposed melanoma samples sequenced in this work. **a** The number of nonsynonymous SNVs and indels per melanoma exome (columns), cohort, age, frequently mutated genes in either subtype (Methods), nonsynonymous base substitution frequencies, and dominant COSMIC mutational signatures^122, 123^. Sig. signature, 5mC 5-methylcytosine. See also Supplementary Data 2b, c for the results of mutational significance analysis with MutSigCV^102^. **b** The number of significant focal amplifications and deletions (GISTIC *Q* < 0.05) per melanoma exome (columns), ordered identically to panel **a**. Cytobands with focal amplifications or deletions with at least 10% recurrence frequency in either melanoma subtype are shown (GISTIC *Q* < 10^–5^), ordered by the relative difference in recurrence frequency in acral versus sun-exposed melanoma. **c** Genes are plotted according to the fraction of acral (*y*-axis) or sun-exposed (*x*-axis) tumors where they are present with ≥4 copies. Considering the genome-wide distribution of differences in recurrence frequencies between melanoma subtypes, genes are identified as significantly recurrent in acral or sun-exposed melanomas if their |*z*-score | ≥ 3 (dashed lines). Significantly recurrent genes are colored according to their cytoband location (inset). For clarity, a small amount of jitter was added to distinguish overlapping genes. Source data are provided as a Source Data file.

To verify key somatic lesions in acral melanoma, we began by performing a comparative genomics analysis against sun-exposed melanoma. We observed striking variation in the prevalence of SNVs and insertions/deletions (indels) between melanoma subtypes, confirming a nearly tenfold lower mutational burden in acral melanoma^2,13^ (median of 406 vs. 42 nonsynonymous variants per exome in sun-exposed vs. acral melanoma, respectively; *P* = 2.2 × 10^–6^, two-sided, unpaired Wilcoxon rank-sum test; Fig. 1a, Supplementary Data 2a–c, and Supplementary Data 3). The most commonly mutated genes in acral melanomas were RAS family members (22% in *NRAS*, *KRAS*, and *HRAS*), followed by *KIT* (15%), *BRAF* (8%), and *TP53* (4%). The recurrence frequencies were similar between cohorts (Supplementary Table 1). Mutational signature analysis^17^ corroborated the prevalence of UV-induced mutagenesis in sun-exposed melanomas. In contrast, mutational signatures in acral melanomas were largely attributable to deamination of 5-methylcytosine (signature 1) (which can arise from reactive oxygen species during melanin synthesis^30^), as well as alkylating agents (signature 11) and APOBEC activity (signature 13) (Fig. 1a, bottom and Supplementary Data 3).

As expected, focal amplifications were a core feature of acral melanoma in both cohorts (median of 20 vs. 11 per exome in acral vs. sun-exposed melanoma, respectively; *P* = 1.24 × 10^–10^, two-sided, unpaired Wilcoxon rank-sum test; Fig.1b, top; Supplementary Figs. 1e,2a and Supplementary Data 4). Among highly recurrent gene-level amplifications with at least four copies, those in chromosomes 4, 5, 8, 11, 12, and 22 were nearly exclusive to acral melanomas in our study (Fig. 1c and Supplementary Data 4). The most common amplifications enriched in acral melanoma were in cytobands 11q13.3 (47%), 5p15.33 (42%), 8q24.3 (42%), 20q13.33 (40%), 7p22.3 (39%), 22q13.1 (39%), and 22q11.21 (38%) (Fig. 1b, Supplementary Fig. 2a, and Supplementary Data 4). Recurrent focal deletions, which included alterations in known genes such as *CDKN2A* (9p21.3)^22,31,32^, were less prevalent than in sun-exposed cases (Fig.1b, bottom; Supplementary Fig. 2b, and Supplementary Data 5). Amplification and deletion frequencies were largely maintained in both acral cohorts (Supplementary Data 4,5). We also identified multiple fusion genes with potential roles in oncogenesis, including several not previously described in acral melanoma (Supplementary Data 6).

Collectively, these results provide a comprehensive resource of somatic lesions in acral melanomas from genetically-distinct patient populations; corroborate and extend previous genomic studies^2,4,7–15^, and demonstrate the integrity and high quality of our data for downstream clinical analysis.

### Somatic determinants of risk in acral melanoma

**Fig. 2:**
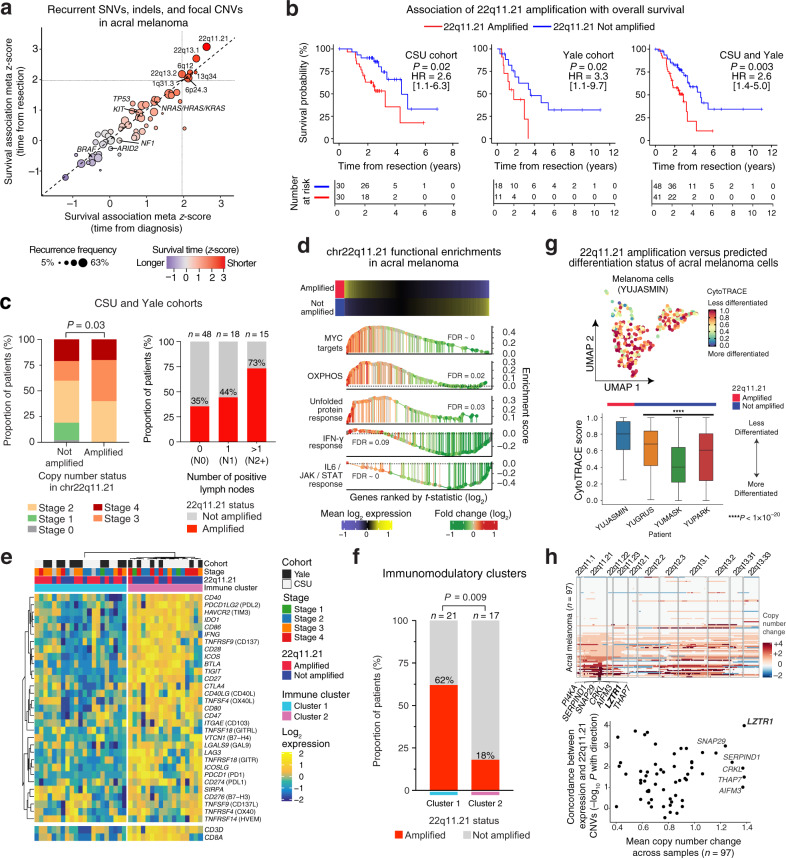
Focal amplifications in 22q11.21 are linked to shorter survival time, regional metastasis, and depletion of immunomodulatory programs in acral melanoma. **a** Association between recurrent somatic alterations and overall survival in acral melanoma. Shown are genes with a nonsynonymous mutation frequency ≥5% in either melanoma subtype and focal copy number events with ≥10% recurrence frequency in each cohort. |*Z* | > 1.96 is *P* < 0.05. **b** Overall survival of acral melanoma patients stratified by 22q11.21 amplification status. Significance was assessed with a two-sided log-rank test. HR hazard ratio. 95% HR confidence intervals are shown in brackets. **c** Left: Acral melanoma stage versus 22q11.21 amplification status. Significance was evaluated by a Chi-square test. Right*:* Fraction of 22q11.21*-*amplified melanomas versus lymph node status. **d** Hallmark pathways enriched in 22q11.21-amplified vs. non-amplified acral melanomas, as determined by pre-ranked gene set enrichment analysis. OXPHOS oxidative phosphorylation. **e** Hierarchical clustering of 31 immunomodulatory genes (average linkage with Euclidean distance) in acral melanomas. *CD3D* and *CD8A* are lineage markers for T cells and CD8 T cells, respectively. **f** Frequency of 22q11.21-amplified acral melanomas in immune clusters 1 and 2 from **e**. Significance was determined by Fisher’s exact test. **g** Top: UMAP showing CytoTRACE-inferred differentiation scores of acral melanoma cells from a 22q11.21-amplified specimen (YUJASMIN). Bottom: CytoTRACE scores (acral melanoma cells) in amplified vs. non-amplified tumors. Box center lines, bounds of the box, and whiskers indicate medians, first and third quartiles, and minimum and maximum values within 1.5×IQR (interquartile range) of the box limits, respectively. Significance was determined using a two-sided, unpaired Wilcoxon rank-sum test relative to YUJASMIN (*n* = 312 cells) for YUGRUS (*n* = 3786 cells, *P* = 1.21 × 10^–22^), YUMASK (*n* = 15,006 cells, *P* = 4.38 × 10^–87^), and YUPARK (*n* = 8141 cells, *P* = 1.06 × 10^–35^). **h** Top: CNVs within the 22q arm of 97 acral melanomas. Genes within the minimum region of focal amplification in 22q11.21 are indicated. Columns indicate genes ordered by location. Bottom: Mean gene-level copy number change in 97 acral melanomas versus the concordance between expression and copy number for each gene. The latter is expressed as the –log_10_ *p* value of a two-sided, unpaired Wilcoxon rank-sum test. Negative associations with amplification status were multiplied by –1. Only genes within the 22q11.21 focal amplification identified by GISTIC are shown (*n* = 24; Supplementary Table 6). Source data are provided as a Source Data file.

Having systematically cataloged somatic aberrations in nearly 100 acral melanomas, we next sought to evaluate their clinical significance. We began by focusing on amplification events owing to their unique prevalence in this disease (Fig. 1b). Starting with the most statistically-significant peaks detected by GISTIC^33^ in a pooled analysis of both acral cohorts (*Q* < 10^–5^), we identified several loci linked to adverse overall survival, including peaks involving cytobands 22q11.21 and 22q13.1. Among them, cytoband 22q11.21 was most strongly associated with inferior overall survival (adjusted *P* < 0.05, univariable Cox regression of time from tumor resection; Supplementary Fig. 3a and Supplementary Data 7). This result was maintained when expanding the analysis to include (1) all focal events identified by GISTIC (*Q* < 0.05) with at least 10% recurrence frequency in each acral cohort and (2) all genes with a nonsynonymous mutation frequency of at least 5% in either melanoma subtype (Fig. 2a and Supplementary Data 7). We also considered focal amplifications identified from the largest cohort (CSU) and tested in each cohort separately (Fig. 2b and Supplementary Fig. 3b). Again, 22q11.21 amplification was a leading determinant of adverse survival.

Given this observation, we sought to better understand the clinical phenotype of 22q11.21 focal amplification. We first tested whether 22q11.21 is a surrogate for the advanced disease at the time of tumor resection. Intriguingly, 22q11.21 amplifications were observed across all stages except stage I disease (*P* = 0.03, Chi-square test; Fig. 2c, left). We verified this result in three independent acral melanoma cohorts, including an external dataset comprised of 33 patients for whom stage at presentation was known^11^, demonstrating that 22q11.21 amplification is likely a late-arising event in acral melanoma (Supplementary Fig. 3c). Given this result, which was not attributable to tumor purity (Supplementary Fig. 3d, e), we reassessed survival associations using stage as a covariate. Regardless of whether we examined all patients or just those with stage II through IV disease, 22q11.21 amplifications remained significant after bivariable adjustment for stage (*P* = 0.008 and 0.024, respectively; Cox proportional hazards regression). This was also true for acral patients with advanced disease (III or IV) (*P* = 0.03, Cox proportional hazards regression; Supplementary Fig. 3f), for whom stage alone did not significantly stratify outcomes. We observed similar results when controlling for other potential confounding variables, including tumor-infiltrating lymphocyte (TIL) content and copy number burden per sample (Supplementary Tables 2, 3). Moreover, when evaluating CNVs detected by alternative genotyping algorithms, the significance of 22q11.21 amplification status in multivariable models was maintained (Supplementary Table 4).

As a common late-arising event, we next tested if 22q11.21 amplifications might correlate with tumor progression. Indeed, in both acral cohorts, we observed a strong positive correlation between 22q11.21 amplification frequency and the number of positive lymph nodes per patient (Fig. 2c, right and Supplementary Fig. 3g). Remarkably, nearly 75% of patients with >1 positive lymph node harbored at least one additional focal copy of 22q11.21 (Fig. 2c, right). Reanalysis of WES data from an independent study^11^ confirmed this trend (Supplementary Fig. 3h). While this association was observed in both primary and metastatic tumor specimens, the latter showed a modest but consistent increase in amplification frequency after controlling for lymph node status (Supplementary Fig. 3i). No other associations with clinical indices were observed (Supplementary Fig. 3j and Supplementary Data 1).

Taken together, these data reveal that 22q11.21 focal amplification is a conserved, predominantly late-arising somatic event linked to poor survival and regional metastasis in acral melanoma, independent of White or Asian ancestry. Accordingly, this event could represent a critical step in the initiation or maintenance of nodal metastasis.

### Integrative genomics of 22q11.21 focal amplification

To explore the biological significance of 22q11.21 amplification in acral melanoma, we next examined transcriptional hallmarks of 22q11.21-amplified tumors. By employing a linear model adjusted for stage (Methods), we rank-ordered genes by their differential expression in 22q11.21-amplified tumors and performed gene set enrichment analysis^34^ (Fig. 2d). Overall, 22q11.21-amplified melanomas were significantly enriched in canonical signaling pathways associated with tumorigenesis and metabolic activity, including MYC target genes, oxidative phosphorylation, and unfolded protein response^35^. In contrast, patients with non-amplified tumors showed higher expression of immunoreactive programs such as IL6/JAK/STAT and IFN-γ response pathways. We hypothesized that such patients might be superior candidates for existing or emerging immunotherapies (Supplementary Fig. 4). Consistent with this possibility, we observed a striking reciprocal relationship between 22q11.21 amplification and the expression of immunomodulatory genes, including key targets of immune checkpoint blockade (e.g., *PDCD1*, *CTLA4*) (Fig. 2e). Among patients with high expression of immunomodulatory genes, only 12% were amplified, whereas, among patients with low expression, 62% were amplified (Fig. 2f). This result was statistically significant (*P* = 0.009, Fisher’s exact test), indicating that 22q11.21-amplified and non-amplified tumors enrich for “cold” and “hot” tumor microenvironments, respectively. Furthermore, by expression deconvolution analysis^36^, we observed a distinct trend toward higher immune content in non-amplified tumors, including a notable enrichment of follicular helper T cells, which are known to express co-inhibitory and co-stimulatory molecules^37^ (Supplementary Fig. 4).

Next, to extend our analysis to single melanoma cells, we applied single-cell RNA sequencing (scRNA-seq) to four acral melanoma tumor specimens, one with four additional copies of 22q11.21, as determined by WES, and three without 22q11.21 amplification (Supplementary Fig. 5a, b, Supplementary Table 5, and Supplementary Data 3). Using canonical marker genes and copy number inference, 27,332 single-cell transcriptomes were confidently identified as melanoma cells (Supplementary Fig. 5c, Methods). Within the amplified patient (YUJASMIN), we confirmed overexpression of genes on the 22q arm, consistent with WES (Supplementary Fig. 5c, d). Furthermore, by comparing malignant cells between 22q11.21-amplified and non-amplified tumors, we identified amplification-enriched pathways with a striking similarity to those observed in bulk tumors, including oxidative phosphorylation and MYC targets, confirming their malignant origin (Supplementary Fig. 5e).

Immature cancer cells often display elevated metabolism via oxidative phosphorylation and MYC activity^38^ and stemness features in melanoma tumors have been linked to poor survival^39–42^. To test whether 22q11.21-amplified cells exhibit an immature cellular phenotype, we employed CytoTRACE, a recently described in silico method for predicting developmental potential on the basis of single-cell transcriptional diversity^43^. Indeed, cells with 22q11.21 amplification were predicted to be less mature (Fig. 2g). Moreover, we obtained similar results when repeating this analysis with a method based on single-cell entropy signaling^44^ and by evaluating the differential expression of pluripotency-associated genes^45^ (Supplementary Fig. 5f, g). This result was also independent of genes physically located on 22q, implying that 22q11.21-amplified cells exhibit a more accessible genome, a hallmark of immature cells in normal tissues^43^.

Finally, to nominate genes within 22q11.21 for functional analysis, we examined the minimal common region of focal amplification (Fig. 2h, top and Supplementary Fig. 6a). In doing so, we identified six genes, including *LZTR1* (leucine zipper-like transcription regulator 1), which exhibited the greatest copy number change across acral melanoma specimens, on average (Fig. 2h, bottom). Remarkably, by ranking genes in 22q11.21 according to their expression in amplified versus non-amplified tumors, *LZTR1* again emerged as the top gene (Fig. 2h bottom, Supplementary Fig. 6a, and Supplementary Table 6). We were struck by this result since LZTR1, a member of the Kelch-like (KLHL) family and an adapter for Cullin 3 (*CUL3*) ubiquitin ligase complexes^46,47^, is considered a tumor suppressor in schwannoma and glioblastoma^46,48,49^. Nevertheless, we found that high expression of *LZTR1* is predictive of poor outcome, both in acral and sun-exposed melanomas from this study, and in 443 advanced sun-exposed melanomas profiled by TCGA (The Cancer Genome Atlas) (Supplementary Fig. 6b–d, Methods). Beyond *LZTR1*, we noted that *CRKL* (CRK like proto-oncogene, adapter protein), a recurrently amplified gene in multiple carcinomas^50–53^, including non-small cell lung cancer (3–13% of cases)^50–52^, was also present in the minimal common region of focal amplification (Fig. 2h). Given these results, we set out to characterize the biological functions of these genes to determine which, if any, underlie the observed clinical phenotype of 22q11.21 amplification.

### Suppression of LZTR1 attenuates melanoma cell proliferation and induces apoptosis independent of Ras or MAPK activity

**Fig. 3:**
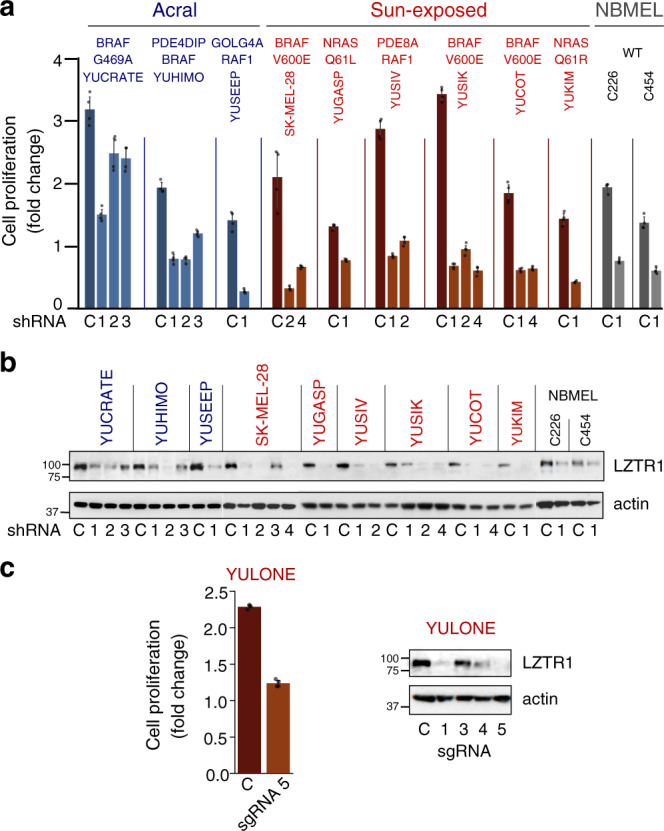
Cell proliferation in response to the suppression of LZTR1. **a** Impact of LZTR1 knockdown on cell proliferation in nine primary melanoma cell lines and two normal human melanocyte lines (NBMEL). Key mutations are indicated (Supplementary Table 7). Bar plots depict mean fold change between the 3rd and 6th day after infection with *LZTR1* shRNA (numbered), as compared to control (scrambled) shRNA (C) with error bars indicating 95% confidence intervals. **b** Western blots with anti-LZTR1 antibodies showing the efficacy of LZTR1 knockdown, related to panel **a**. This experiment was reproduced twice with LZTR1 shRNA 1 and shRNA 2, compared to control. **c** Cell proliferation (left) and LZTR1 expression (right) of a sun-exposed melanoma cell line that lost one *LZTR1* allele in response to genomic modification by different CRISPR-Cas9 sgRNAs targeting *LZTR1*. Bar plots depict fold change between the 3rd and 6th day after infection with sgRNA 5. Data represent one experiment. Bars in **a**, **c** represent the mean of triplicate wells with error bars indicating 95% confidence intervals. Actin levels in **b** and **c** show protein loading. Cell lines are indicated above all plots in **a**–**c** and colored according to their origin: acral melanoma (blue), sun-exposed melanoma (red), normal melanocyte (gray). NBMEL newborn melanocytes, WT wildtype. Source data are provided as a Source Data file.

We began by silencing several 22q11.21-amplified genes using lentiviral delivery of short hairpin RNAs (shRNAs) (Supplementary Data 8), with the goal of determining the impact of targeted knockdowns on melanoma cell proliferation. Treatment of acral melanoma cell lines with *CRKL* shRNA induced growth arrest, but only two of five tested cell lines were highly affected (Supplementary Fig. 7a). Conversely, silencing of *LZTR1* consistently arrested cell proliferation. This was the case regardless of subtype (acral or sun-exposed) or mutations in *BRAF* or *NRAS* (Supplementary Fig. 7a, b). In addition, we observed growth arrest in normal melanocytes derived from two independent foreskins (Fig. 3a, b). We ruled out off-target effects because four different *LZTR1*-directed shRNAs induced growth arrest, as did CRISPR-Cas9 sgRNA directed against *LZTR1* (Fig. 3a–c; Supplementary Fig 7b; and Supplementary Data 8). The observed phenotype had a long-term effect since *LZTR1-*null melanoma cells did not survive in vitro, whereas cells infected with control shRNA (scrambled) continued to proliferate. We also tested the depletion of *SNAP29* and *THAP7*, both of which are located within the minimum common region of 22q11.21 focal amplification (Fig. 2h top and Supplementary Table 6). Knockdown of these genes had little to no effect on proliferation (Supplementary Fig. 7c, d).

**Fig. 4:**
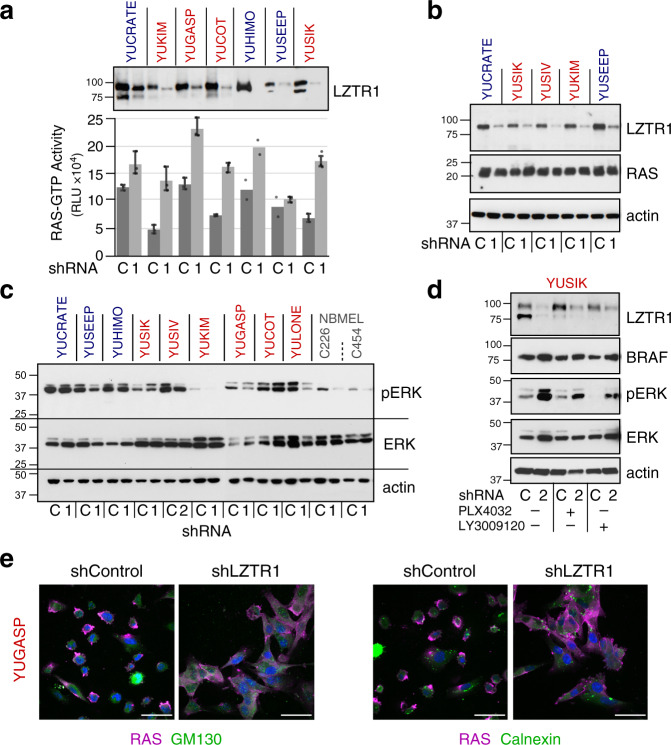
Changes in cell signaling in response to LZTR1 knockdown. **a**, **b** Impact of LZTR1 loss on **a** RAS-GTP activity as measured by RAS-GTPase activation ELISA assay, and **b** RAS levels, 5 days after *shLZTR1* infection. Data in **a** are expressed as the average of triplicate or duplicate wells with error bars indicating 95% confidence intervals. LZTR1 knockdown showed a statistically significant increase of RAS-GTP activity (*P* = 0.006; two-sided, unpaired Wilcoxon rank-sum test). **c** Effect of LZTR1 loss on MAPK activity. **d** YUSIK melanoma cells were incubated with the PLX4032 (500 nM) or LY3009120 (100 nM), BRAF, and pan-RAF kinase inhibitors, respectively, for 4 h at the end of treatment with *LZTR*1 shRNA. **e** RAS translocation to the cytoplasm in response to *shLZTR1*. RAS is visualized by staining with magenta, and GM130 or calnexin with green (Cy2). Scale bar = 50 µm. shRNAs are indicated in panels **a**–**d** by numeric identifiers; C scrambled shRNA control. Actin levels in **a**–**d** show protein loading. Cell lines indicated above the plots are colored according to their origin: acral melanoma (blue), sun-exposed melanoma (red), and normal melanocytes (gray). NBMEL newborn melanocytes. Panels **a**, **b**, **d,** and **e** represent a single experiment; panel **c** represents one of two experiments. Source data are provided as a Source Data file.

Given these results, we sought to better understand the biological consequences of LZTR1 knockdown. Inactivating germline mutations in LZTR1 are associated with Noonan syndrome and functional studies have linked LZTR1 inactivation to RAS ubiquitination, increased RAS-MAPK signaling, and cell proliferation^54–59^. Indeed, suppression of LZTR1 in melanoma cells increased the constitutive levels of GTP-bound RAS, an effect similar to that observed in growth factor-stimulated cells^57,60^. RAS-GTP levels increased in *NRAS*- or *BRAF*-mutant melanoma cells without a change in total RAS protein (Fig. 4a, b).

We also observed changes in MAPK signaling following LZTR1 knockdown. For example, there was an increase in pERK in melanoma cells carrying *BRAF*^*G469A*^ (YUCRATE) or *BRAF*^*V600E*^ (YUSIK and YUCOT) (Fig. 4c). In contrast, pERK decreased in *NRAS*^*Q61L/R*^ melanoma cells (YUKIM and YULONE), *GOLG4A*-*RAF1* or *PDE8A*-*RAF1* fusion-bearing melanoma cells (YUSEEP and YUSIV), and normal human melanocytes (NBMEL) (Fig. 4c). ERK activation was likely due to an increase in BRAF levels (Fig. 4d), enhancing BRAF activity. Treatment of melanoma cells with *BRAF*^*V600E/K*^ or pan-RAF inhibitors (PLX4032 or LY3009120) reduced *shLZTR1*-induced pERK activation (Fig. 4d), rendering further support for the role of BRAF kinase activity. On the other hand, ERK inhibition in *shLZTR1*-treated cells could potentially arise from RAS translocation to the cytoplasm (Fig. 4e), and the consequent disassociation from its membrane-bound mitogenic effectors, which are critical for *NRAS*^*Q61/L/R*^ mutant and WT cells lacking *BRAF* mutations. RAS translocation was not linked to de-ubiquitination, because loss of LZTR1 did not change the levels of ubiquitinated RAS (Supplementary Fig. 8a). Thus, downregulation of LZTR1 induces growth arrest independently of ERK activity, the presence of *BRAF* or *NRAS* oncogenes, and changes in RAS-GTP levels.

**Fig. 5:**
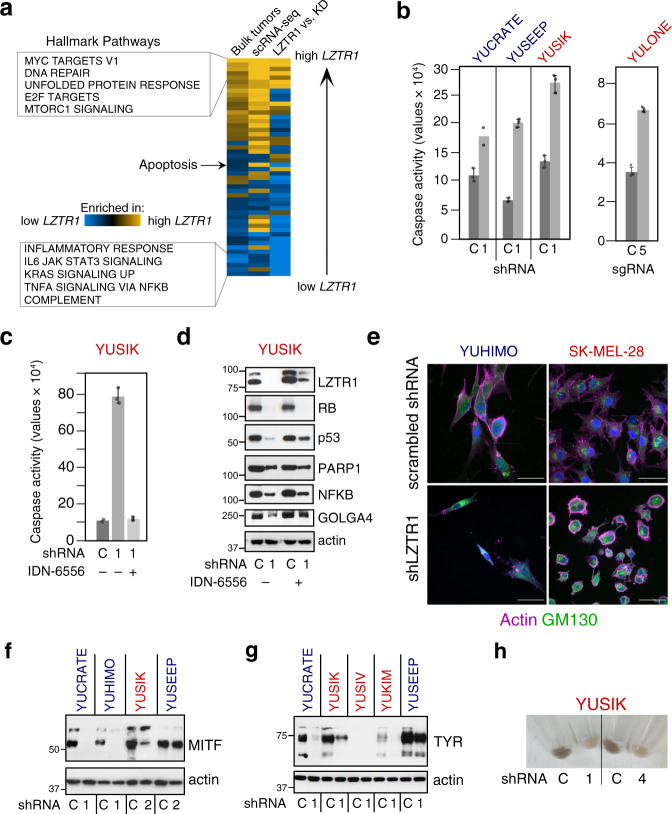
Impact of LZTR1 knockdown on apoptosis and pigmentation. **a** Gene set enrichment analysis (GSEA)^34^ showing concordance in hallmark pathways among bulk acral melanoma tumors (same as Supplementary Fig. 5e), acral melanoma single-cell transcriptomes (scRNA-seq; same as Supplementary Fig. 5e), and a primary melanoma cell line (*LZTR1* vs. KD), in relation to high vs. low *LZTR1* expression. All pairwise comparisons are statistically significant by Spearman correlation (nominal *P* ≤ 0.0005) except for “scRNA-seq” vs. “LZTR1 vs. KD”. Gold, positive normalized enrichment score (NES); blue, negative NES; KD knockdown. **b** LZTR1 shRNA and sgRNA (CRISPR-Cas9) induce apoptosis in melanoma cells. Data were expressed as the average of triplicate or duplicate wells with error bars indicating 95% confidence intervals (CI). **c**, **d** Effects of inhibiting caspase activity with IDN-6556 (IDN, 2 µM, 3 days). As shown in **c**, IDN-6556 suppressed *shLZTR1*-induced caspase activity. Data in **c** are expressed as the average of triplicate (shRNA only and shRNA + IDN-6556), or duplicate (control) wells with error bars denoting 95% CI. As shown in **d**, IDN-6556 increased the levels of LZTR1 (known to be degraded by caspases) and rescued caspase substrates, such as GOLGA4, p53, and to a lesser extent, NF-κB. **e** Effect of *shLZTR1* on cell morphology and actin filament organization. Actin filaments were visualized by staining with rhodamine-phalloidin (magenta) and the Golgi with anti-GM130 (green, Cy2). The nuclei are stained with DAPI (blue). Scale bar = 50 µm. **f**–**h** Impact of *shLZTR1* on MITF (panel **f**), tyrosinase (TYR) (panel **g**), and pigmentation (panel **h**). shRNAs in **b**–**d** and **f**–**h** are indicated by unique numerical identifiers. C scrambled shRNA control. Actin levels in **d**, **f** and **g** show protein loading. Cell lines are indicated above all plots in **b**–**h** and colored according to their origin: acral melanoma (blue), sun-exposed melanoma (red). Panel **e** represents one of two experiments. Source data are provided as a Source Data file.

We next explored if our in vitro melanoma systems effectively recapitulate key 22q11.21-related signaling pathways observed in vivo. To this end, we performed bulk RNA sequencing of a melanoma cell line (YUSIK) to assess the impact of *LZTR1* knockdown. Remarkably, depletion of *LZTR1* induced transcriptome-wide changes that largely mirrored those observed in bulk tumors and single melanoma cells (Fig. 5a).

We noticed that among altered transcriptional programs, apoptosis-related genes were elevated in cell lines and tumors with lower *LZTR1* expression (Fig. 5a). These data are supported by an increase in caspase activity after treatment with *LZTR1* shRNA or sgRNA (Fig. 5b, c), which led to the degradation of known caspase substrates^61^, including pRb, p53, PARP1, NFKB1, and GOLGA4 (Fig. 5d). Notably, GOLGA4 localizes to the Golgi apparatus, the subcellular site of LZTR1^62^. Moreover, *shLZTR1*-induced caspase activity was suppressed by the pan-caspase inhibitor IDN-6556 (Emricasan), which also rescued several substrates, including LZTR1 (Fig. 5c, d). These data are consistent with a previous report showing that LZTR1 undergoes caspase-mediated degradation^62^. Furthermore, *shLZTR1* led to disruptions of cellular organization, including actin depolymerization into irregular shapes (Fig. 5e, left), or the formation of actin rings around the Golgi and nucleus (Fig. 5e, right). Such changes are characteristic of cells undergoing fast or slow apoptotic death, respectively^63^.

Several cell cycle proteins were also downregulated, in line with pathway enrichment analyses (Supplementary Fig. 8b–e). In addition, a major reason for growth arrest in some melanoma cell lines is the downregulation of MITF, a lineage-specific transcription factor critical for melanocyte and melanoma cell proliferation^64^. MITF stability is reduced when phosphorylated by MAPK or KIT^65,66^, and this process was clearly observed in three out of four melanoma cell lines with increased ERK activity (Fig. 5f, as compared to Fig. 4c, d). Downregulation of MITF, as expected, is associated with a decrease in tyrosinase, the key enzyme in melanin synthesis as well as cellular pigmentation (Fig. 5g, h). These results are consistent with our published observations using the same melanoma cell lines^67^.

### Overexpression of LZTR1 in normal melanocytes confers properties of malignant transformation and metastasis

**Fig. 6:**
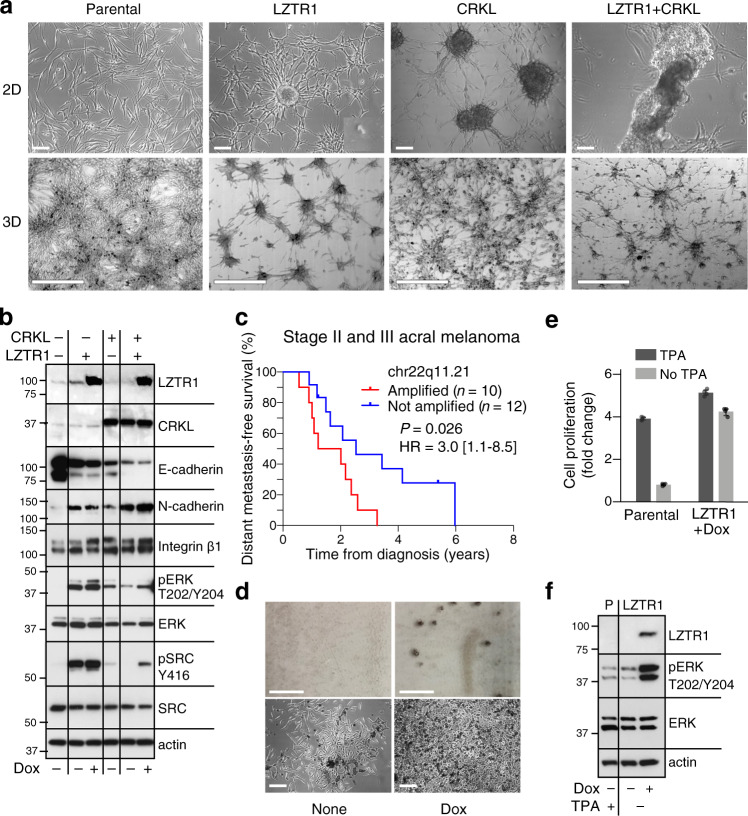
Overexpression of LZTR1 and CRKL confer properties consistent with malignant cell transformation and metastasis initiation. **a** Morphological changes and spheroid formations in early passage normal human melanocytes (NBMEL C1220) overexpressing LZTR1 and/or CRKL. Top: Phase-contrast images of parental and infected cells in 2D culture. LZTR1 images were taken after 2 days of induction with doxycycline (200 ng/ml), CRKL after 3 days of infection with PLX304-CRKL and LZTR1 + CRKL after 6 days infection of LZTR1 melanocytes with PLX304-CRKL and 3 days stimulation with doxycycline. Non-induced LZTR1 cells grew as a monolayer, as seen for the parental non-transformed melanocytes (Parental). Scale bar = 100 µm. Bottom: 3D cultures of melanocytes seeded in 0.5% collagen for 3 days. LZTR1 and CRKL, both alone and in combination, induced aggregation and multicellular spheroids. Scale bar = 500 µm. **b** Western blot showing changes in normal human melanocytes overexpressing LZTR1, CRKL, or both (top two lanes) compared to parental (–). Cells were harvested after incubation in regular medium, or medium supplemented with doxycycline for 2 days when applicable (Dox, 200 ng/ml). Of note, an increase in LZTR1 produced by basal promoter activity was sufficient to induce constitutive MAPK and SRC activities. Actin levels show protein loading. **c** Differences in distant metastasis-free survival (DMFS) between acral melanoma patients stratified by 22q11.21 amplification status. Patients with stage II or III disease at diagnosis with available DMFS data were shown (Yale cohort). Statistical significance was assessed by a two-sided log-rank test. HR hazard ratio. 95% HR confidence interval is shown in brackets. **d** 2D cultures of spontaneously immortalized mouse melanocytes (C57BL) forming colonies in the absence of TPA in response to LZTR1 (Dox). Dark pigmented colonies are seen without magnification (top) and by phase-contrast microscopy (bottom). Scale bar = 100 µm (top) and 200 µm (bottom). **e** Proliferation of parental and LZTR1-transformed C57BL mouse melanocytes. Bar plots represent the mean fold change of *n* = 4 replicates with 95% confidence intervals indicated. **f** Western blots displaying LZTR1 expression and MAPK activation (pERK) in TPA-starved (–) mouse melanocytes in response to doxycycline (+, 200 ng/ml), compared to parental, non-transformed cells (P). Panel **a** represents one of three experiments. Source data are provided as a Source Data file.

We next evaluated the impact of overexpressing LZTR1 in normal melanocytes and compared the effects to overexpression of CRKL. The latter is an SH3/SH2 adapter protein that promotes lung cancer cell invasion via ERK activation^68^ and epithelial-mesenchymal transition (EMT) in colorectal and pancreatic carcinomas^69^. Early passage human melanocytes (passage 4) were transduced with HA-tagged *LZTR1* cloned into the pInd20 lentiviral vector, V5-tagged *CRKL* inserted into the PLX304 vector or both constructs. Overexpression of these genes did not enhance the rate of cell proliferation; rather, melanocytes overexpressing *CRKL* grew slower compared to parental cells (Supplementary Fig. 9a). Nevertheless, within 2–3 days after infection, we noticed a striking induction of anchorage-independent growth, observed as cells overexpressing LZTR1 or CRKL formed three-dimensional clusters in 2D and 3D collagen cultures (Fig. 6a, top and bottom rows, respectively). Moreover, this result—which was reminiscent of a malignant cell phenotype^70^—was further enhanced when both genes were co-expressed (LZTR1 + CRKL), leading to the formation of spheroids that detached from the surface of the dish (Fig. 6a).

During metastasis, primary melanoma cells detach from the dermis and migrate to secondary sites through increased cell–cell interactions and promotion of cancer cell survival. We, therefore, examined changes in adhesion proteins affecting cell–matrix and cell–cell interactions known to mediate aggregation, the formation of spheroids^70^, and in vivo EMT^71,72^. Our data show that E-cadherin was downregulated whereas N-cadherin and integrin β1 were upregulated in response to increased expression of *LZTR1* and *CRKL*, a process that was enhanced when both genes were co-expressed (Fig. 6b). Notably, our results with *CRKL* were consistent with HCT116 colon cancer cells, in which loss of *CRKL* was found to increase E-cadherin expression and shift the cells toward an epithelial phenotype^69^. Importantly, LZTR1 and CRKL, both alone and in combination, induced high levels of constitutively active ERK and SRC relative to parental cells (Fig. 6b, pERK and pSRC), functions that support viability and proliferation. We also identified the downregulation of MITF in cells overexpressing CRKL as the possible cause for growth rate attenuation (Fig. 6b and Supplementary Fig. 9a). Consistent with this finding, while higher expression of MITF defines a proliferative subtype of melanoma (MITF^high^–AXL^low^), lower expression is preferentially associated with invasion (MITF^low^–AXL^high^)^73^.

Based on these findings, we hypothesized that co-amplification of *LZTR1* and *CRKL* might lead to increased rates of distant recurrence. Given that 22q11.21 amplification is preferentially a late-arising event in acral melanoma (Fig. 2c, left), we tested this hypothesis by examining acral melanoma patients diagnosed with stage II or III disease. Indeed, in patients for whom distant metastasis-free survival (DMFS) data were available, focal amplification of 22q11.21 was associated with earlier development of distant metastatic disease, with a median lead time of nearly 1 year (Fig. 6c).

Finally, we investigated whether overexpression of LZTR1 or CRKL releases normal human melanocytes from their dependency on growth factors, a common phenotype of metastatic melanoma cells^74^. While normal human melanocytes retained their growth factor dependency (Supplementary Fig. 9), LZTR1, but not CRKL, enabled immortalized mouse melanocytes to form colonies and divide in the absence of their only required growth factor, TPA (tetradecanoyl phorbol acetate)^75^ (Fig. 6d, e). This phenotype is likely the consequence of MAPK activation, as seen by the presence of phosphorylated ERK (Fig. 6f).

Taken together, these results strongly implicate *LZTR1* and *CRKL* in malignant transformation and the initiation of metastasis. While both genes showed similar phenotypes, the effects of overexpression were notably enhanced when *LZTR1* and *CRKL* were co-expressed. However, only *LZTR1* released immortalized mouse melanocytes from their dependency on growth factors, a characteristic shared by melanoma cells.

## Discussion

Acral melanoma has a higher incidence in non-White populations compared to other melanoma subtypes, accounting for over 50% of melanomas diagnosed in Asian populations but less than 10% in White populations^29,76–82^. Our work establishes common features of acral melanomas independent of Asian or White ancestry. These include (1) the consistent association between specific focal amplifications and poor outcomes and (2) the identification of *LZTR1* as a key gene within 22q11.21, the most prognostic recurrent alteration identified in both acral cohorts. Based on these findings, we performed a comprehensive analysis of LZTR1 signaling pathways and obtained functional evidence for LZTR1 as a tumor promoter.

*LZTR1* is co-amplified with *CRKL* and downregulation of each gene inhibits melanoma cell proliferation, albeit to varying degrees. While *CRKL* has been linked to tumor growth as a candidate oncogene in several human malignancies, including lung adenocarcinoma^50–52^, *LZTR1* is generally considered a tumor suppressor. Germline mutations in *LZTR1* are involved in Noonan syndrome^56,83^, schwannomatosis^49^, and glioblastoma^46,84^. Moreover, somatic loss-of-function mutations in *LZTR1* occur in 22% of glioblastomas. These mutations drive self-renewal and growth of glioma spheres^46^, consistent with a role in tumor suppression. However, despite these findings, *LZTR1* is amplified in a subset of carcinomas (up to 8.3%), including bladder, uterine, and lung cancers^85,86^. These data, coupled with our results, suggest that *LZTR1* could have tumor-promoting capabilities in multiple human malignancies.

Unique aspects of our study include the broad range of tumor specimens analyzed and the utilization of cells harboring different oncogenes that modulate LZTR1 activity. For example, in *NRAS*-mutant melanoma, RAS mis-localized to the cytoplasm in response to *shLZTR1* and caused MAPK inhibition. On the other hand, the elimination of LZTR1 in BRAF-mutant cells increased BRAF levels, leading to ERK activation. In several cell lines, ERK activation induced growth arrest via MITF degradation, a process unique to melanocytes and the melanoma system^65,66^.

Importantly, our study demonstrates that *LZTR1* and *CRKL*—two of the top genes associated with 22q11.21 amplification in acral melanoma—facilitate anchorage-independent growth in normal human melanocytes, likely by reducing E-cadherin, increasing N-cadherin, and activating integrin β1. While the reciprocal expression of E-cadherin and N-cadherin in early melanoma progression has been known for about two decades^71,87^, our findings specifically link these events to the modification of two genes. In addition, we observed activation of MAPK and SRC kinases, the likely consequences of integrin signaling^88,89^. The ability of LZTR1 to convert immortalized mouse melanocytes to a growth-factor independent mode of proliferation, a major characteristic of melanoma cells in culture, further underscores its tumorigenic potential. These results agree with our genomic observation that 22q11.21 amplification is predominantly a late-arising event associated with regional and distant metastasis.

Separately, we identified a striking inverse relationship between immunomodulatory genes and 22q11.21 amplification. It is tempting to speculate that high levels of *LZTR1* reduce the inflammatory response while protecting cells from stress-induced apoptosis, thereby facilitating metastasis. Conversely, patients with low levels of *LZTR1* preferentially harbor a hot tumor microenvironment, which might provide benefit from immunotherapy. Future studies will be needed to explore these possibilities.

In summary, we demonstrate that focal amplifications of cytoband 22q11.21 are a leading determinant of shorter survival time in acral melanoma. Our genomic and functional experiments provide critical insights into the pathogenesis of this disease and strongly implicate LZTR1 as a tumor promoter and promising therapeutic target.

## Methods

### Human subjects

All clinical specimens in this study were collected with informed consent for research use and were approved by the Yale University and Central South University Institutional Review Boards in accordance with the Declaration of Helsinki. No compensation was provided to the participants in this study. CSU samples were collected from Xiangya Hospital, Hospital for Skin Diseases (Institute of Dermatology), Chinese Academy of Medical Sciences in Nanjing, Third Affiliated Hospital of Sun Yat-sen University, Hunan Provincial Tumor Hospital, Xiangya Hospital, Central South University, First Affiliated Hospital of Harbin Medical University, and Wuhan Union Hospital. The melanoma cell lines were from the Specimen Resource Core of the Yale SPORE in Skin Cancer. Melanoma tumor specimens were excised to alleviate the tumor burden. All analyzed tumor specimens were 2 cm × 2 cm × 2 cm on average and were derived from excess surgical material not required for clinical diagnosis or patient care.

### Nucleic acids extraction

Melanomas were sequenced from snap-frozen tumors (Yale and CSU cohorts) or low passage cell cultures (<4) as previously described^2,4^ (Supplementary Data 1, 8). DNA from melanoma cells and freshly frozen tumors was extracted with the DNeasy purification kit (Qiagen Inc., Valencia, CA). High melanin content was removed with *OneStep*™ PCR Inhibitor Removal Kit (Zymo Research Corporation, Irvine, CA). Direct-zol™ RNA MiniPrep w/ Zymo-Spin™ IIC Columns (Zymo catalog no. D4019) were used to extract RNA from tumors, and the RNeasy PowerLyzer Tissue & Cells Kit (Qiagen, catalog no. 15055-50) was used to extract RNA from peripheral blood mononuclear cells (PBMCs) and melanoma cells.

### Whole-exome sequencing

The quality of genomic DNA was determined by estimating the A_260_/A_280_ and A_260_/A_230_ ratios by nanodrop, both of which required to be >1.8, and by electrophoresis in 1% agarose gel in which high-quality DNA migrates as a single high molecular weight band. One µg of genomic DNA was sheared to a mean fragment length of about 220 bp using focused acoustic energy (Covaris E220). The size distribution of the fragmented sample was determined by using the Caliper LabChip GX system. The fragmented DNA samples were transferred to a 96-well plate and library construction was completed using a liquid handling robot. Following fragmentation, we added T4 DNA polymerase and T4 polynucleotide kinase that blunt end and phosphorylate the fragments. The large Klenow fragment then adds a single adenine residue to the 3′ end of each fragment and custom adapters (IDT) are ligated using T4 DNA ligase. Magnetic AMPure XP beads (Beckman Coulter) were used to purify and size select the adapter-ligated DNA fragments. The adapter-ligated DNA fragments were then PCR amplified using custom-made primers (IDT). During PCR, a unique six-base index was inserted at both ends of each DNA fragment. Sample concentration was determined by picogreen and the fragment length distribution using the Caliper LabChip GX system. Samples yielding at least 1 µg of amplified DNA were used for capture.

For the CSU cohort, targeted capture was performed using the NimbleGenSeqCap Med Exome 47 M kit, followed by 151 bp paired-end sequencing on the Illumina HiSeq X Ten platform. TrimGalore (v0.3.7) and FastQC (v0.11.2) were used to remove adapters and low-quality sequences from the raw data. For the Yale cohort, equal amounts of each sample were pooled prior to capture. For example: for 16 samples per lane 62.5 ng of each genomic DNA library was pooled (1 µg total) and lyophilized with Cot-1 DNA and universal adapter blocking oligos (IDT). The dried sample was reconstituted according to the manufacturer’s protocol (IDT), heat-denatured, and mixed with biotinylated DNA probes produced by IDT (xGen Exome Panel). Hybridizations were performed at 65 °C for 16 h. Once the capture was complete, the samples were mixed with streptavidin-coated beads and washed with a series of stringent buffers to remove nonspecifically bound DNA fragments. The captured fragments were PCR amplified and purified with AMPure XP beads. Samples were quantified by qRT-PCR using a commercially available kit (KAPA Biosystems) and insert size distribution was determined with the LabChip GX. Samples with a yield of ≥0.5 ng/µl were used for sequencing. Sample concentrations were normalized to 2 nM and loaded onto Illumina NovaSeq 6000 flow cells at a concentration that yields at least 600Gbp of passing filter data per lane. Samples were sequenced using 101 bp paired-end sequencing reads according to Illumina protocols.

### Bulk RNA sequencing

For the CSU cohort, total RNA was depleted of rRNA using the Ribo-Zero rRNA removal kit, with 1 μg of total RNA used as input for rRNA removal. Sequencing libraries were generated using the TruSeq RNA sample prep kit (Illumina). The libraries were sequenced as 151 bp paired-end reads using an Illumina HiSeq X Ten platform. For the Yale cohort, rRNA was depleted starting from 25–1000 ng of total RNA using the Kapa RNA HyperPrep Kit with RiboErase (KR1351). Indexed libraries that met appropriate cut-offs for both quantity and quality were quantified by qRT-PCR using a commercially available kit (KAPA Biosystems) and insert size distribution was determined with the LabChip GX or Agilent Bioanalyzer. Samples with a yield of ≥0.5 ng/ul were used for sequencing. Samples were run on a combination of Illumina HiSeq 2500, HiSeq 4000, and NovaSeq instruments, and multiplexed using unique dual barcode indexes (to avoid sample contamination and barcode hopping).

### Single-cell RNA sequencing

To obtain single-cell transcriptional portraits of 22q11.21-amplified and non-amplified tumors, we analyzed a primary acral melanoma specimen (YUJASMIN) with six focal copies of 22q11.21, as determined by WES, and three primary acral melanomas without amplification of 22q11.21 (YUGRUS, YUMASK, YUPARK; Supplementary Data 1). For YUJASMIN, the 10x Chromium 5′ expression profiling platform with V1 chemistry was applied to a cryopreserved tumor cell suspension sorted for viable singlets to target 10,000 cells (Supplementary Fig. 10). Cells were sorted in the following ratios prior to library preparation: 50% CD3^+^CD45^+^ T cells: 25% CD3^–^CD45^+^ non-T immune cells: 25% CD45^–^ stromal/cancer cells. Cell viability was assessed by the LIVE/DEAD™ Fixable Red Dead Cell Stain Kit (catalog no. L34971, Thermo Fisher). The following antibodies were used: Alexa Fluor^®^ 488 anti-human CD45 antibody (clone H130, catalog no. 304019, BioLegend); APC anti-human CD3 antibody (clone HIT3a, catalog no. 300319, BioLegend) (Supplementary Data 9). The 10x library was sequenced on an Illumina HiSeq 2500 instrument. For the other three tumors, the 10x Chromium 5′ assay with V2 chemistry was applied to cryopreserved tumor cell suspensions and sequenced on an Illumina NovaSeq 6000 instrument.

### Tumor genotyping from whole-exome sequencing data

Sequencing reads from exome-captured samples were analyzed with a combination of germline and somatic variant calling, permitting the identification of somatic variants, candidate loss-of-heterozygosity (LOH) regions, and copy number variation (CNV) regions.

BAM files of aligned reads were created for each sample by aligning the sequencing reads to the GRCh37 human reference with decoy sequences (the “hs37d5” reference) using BWA MEM^90^ (v0.7.15), marking duplicates using Picard MarkDuplicates (http://broadinstitute.github.io/picard) (v2.17.11), and then performing indel realignment and base quality score recalibration using GATK v3.2^91^. Then, variants were called using the tumor/normal bam files in three ways: (1) a joint variant call using GATK HaplotypeCaller, GenotypeGVCFs and hard filtering following GATK 3.2 best practices; (2) somatic SNP variant calls using MuTect with options “max_alt_alleles_in_normal_count=6”, “max_alt_allele_in_normal_fraction=0.1” and “max_alt_alleles_in_normal_qscore_sum=200”; (3) somatic indel variant calls using Indelocator with options “minCoverage=6”, “minNormalCoverage=4”, and “minFraction=0.2”. The output from the three variant callers were merged using in-house scripts into a single VCF file, containing the union of GATK variants and MuTect/Indelocator somatic variants, marking variants called as somatic by MuTect or Indelocator as “somatic”.

Those variants were annotated using Annovar^92^ and Variant Effect Predictor (VEP)^93^ (v91), and then the somatic variants were filtered using the following criteria: (1) tumor alt depth ≥4, (2) normal read depth ≥4, (3) normal alt depth ≤1 or normal alt frequency less than 1/5 tumor alt frequency, (4) the maximum population frequency of the variant from ExAC^94^, NHLBI, 1000 Genomes, or Yale Exome database must be less than 2% for a cancer-related gene (any gene in the Oncomine or Foundation Medicine gene panels or COSMIC CG Census gene list) or 1% for any other gene. Also, only protein changing variants with a VEP impact of MEDIUM or HIGH, or variants within 15 bases of a protein-coding splice site were reported in the final output.

Unless stated otherwise, we estimated somatic copy number using a previously published and validated technique^95–99^, referred to as CNVL. Specifically, candidate loss-of-heterozygosity (LOH) regions were identified using the joint variant calls generated from GATK^100^. For each variant in the GATK joint calling, where the normal sample was called heterozygotic and had depth ≥20, the allele frequency difference (AFD) was calculated as “abs (tumorAF – normalAF)”), subtracting tumor from normal allele frequency. Those differences across the genome were smoothed using the R loess and predict (“predict(loess(AFD, span = 0.28))”), and regions ≥0.1 were considered as allelic deviations from the normal. The mean of each region was calculated, and the maximum mean was doubled to provide the tumor purity estimate (as, for example in a LOH region, the deviation from normal heterozygotic calls is one-half the tumor fraction, plus or minus one of the two tumor alleles).

CNV regions were identified by first calculating the mean read depth for each RefGene coding exon, for the tumor and normal samples. Normalized tumor/normal read depth ratios were computed for each exon (normalized by the mean read depth of the tumor and normal across the exome), and then, using partitioning of the genome into 20 kb bins, a mean ratio for each 20 kb region of the genome, which contains an exon, was computed. Those ratios were converted to log-ratios, then de-noised and segmented by circular binary segmentation (CBS) using the DNA copy library from R (http://bioconductor.org/packages/release/bioc/html/DNAcopy.html). Regions with a value deviating from 0.0 are identified as CNVs. For each CNV, the log-2 ratio is converted back to a simple ratio and the copy number is calculated as “2 + round((ratio – 1.0)/step))”, where “step” is one of three empirically determined values based on the estimated tumor purity, specifically 0.5, 0.4, or 0.32 for samples with purity ≥80%, purity between 40 and 80% or purity ≤40%, respectively.

Tumor purities were also estimated using the software ABSOLUTE (v1.0.6)^101^. The CBS segmentation results from the CNV calculation, as well as an MAF file of the somatic variants, were given as input to ABSOLUTE, using most of the default parameters used by https://www.genepattern.org/modules/docs/ABSOLUTE for their example exome data (sigma.p = 0, max.sigma.h = 0.2, min.ploidy = 0.5, max.as.seg.count = 1500, max.non.clonal = 1, max.neg.genome = 0, copy_num_type = ”total”), but setting max.ploidy = 5 and primary.disease = “NA”. Purities estimated by ABSOLUTE and the abovementioned approach (CNVL) were highly concordant across the 231 melanoma exomes profiled in this work (Spearman ρ = 0.89, *P* = 3.8 × 10^–88^; Supplementary Data 1a).

Regions of significant focal amplification and deletion were identified with GISTIC2.0 (v2.0.23, release date 27 Mar 2017)^33^ using the CBS segmentation files described above and the hg19 reference genome (GRCh37). No marker input file was provided. Parameters were specified according to the authors’ recommended run profile: amplification and deletion thresholds were set to 0.1, the *q* value threshold was set to 0.1 with a confidence level of 0.95, and log_2_ ratios were capped at 1.5. Gene-level GISTIC analysis and broad analysis were also applied, with a focal length cutoff of 0.7. Wide peaks identified with a *Q* value less than 0.1 in each melanoma subtype were aggregated into a master list (Supplementary Data 4, 5), and genes within each wide peak were used to construct a copy number matrix. Of note, if two or more peaks were identified within the same cytoband, we appended a suffix to the cytoband name to denote the melanoma subtype in which the cytoband was identified (AC acral; SE sun-exposed). If more than one peak was identified within the same cytoband for a given melanoma subtype, the subtype acronym was followed by a numerical identifier (1, 2, etc.). For cases in which one peak completely encompassed another one and where both peaks had the same orientation (i.e., amplified or deleted), the shorter one was eliminated.

For comparative genomics and survival analyses, we constructed a matrix containing the mean copy number per wide peak (rows) for each melanoma tumor sample (columns). The copy number per wide peak was calculated as the average gene-level copy number, as determined by CNVL, per wide peak. We subtracted 2 from all gene-level values so that copy number-neutral regions are equal to 0 (Supplementary Data 3).

MAF files for each sample’s somatic variants were generated by vcf2maf (v1.6.14), using VEP (v91) and filtering by ExAC (v0.3). The acral variants, and separately the sun-exposed variants, were combined into a single MAF file and analyzed with MutSigCV (v1.41)^102^, which was run using default parameters and the exome coverage, covariate, and dictionary files included in the MutSigCV package (Supplementary Data 2b, c).

### Visualization of somatic alterations across patients

Recurrently mutated genes and significant focal CNVs were visualized using the *Oncoprint* function in *ComplexHeatmap*^103^ (v1.20.0). The default bar plot (top) was replaced with a bar plot showing the number of nonsynonymous SNVs and indels per patient. Genes were included if they are known hotspot genes that subdivide cutaneous melanoma into *BRAF*, *RAS*, and *NF1* subtypes^5^. We added additional genes identified by TCGA to be recurrently mutated in cutaneous melanoma (Fig. 1a in Cancer Genome Atlas Network, *Cell* 2015). We also added *KIT*, a key hotspot gene in acral melanomas^15,18^. For CNV regions, amplifications and deletions were calculated by averaging the GISTIC-generated gene-level copy numbers for all genes within each wide peak. Peaks with an average copy number above 3 (one additional copy) were deemed amplified, whereas peaks with an average copy number below 1.4 were considered deleted. Peaks were included if present in at least 10% of either melanoma subtype.

### Bulk RNA-seq analysis

Raw RNA-seq reads were aligned with Salmon^104^ (v0.99) to the GENCODE v.25^105^ reference transcript assembly. Subsequently, tximport^106^ was used to generate an expression matrix normalized to transcripts per million (TPM). Protein-coding genes were determined using Ensembl release 92 human annotation^107^ (GRCh38.p12, Apr 2018), extracted by biomaRt^108^ (v2.40.5) and non-protein-coding genes were omitted. Expression values were renormalized to TPM after this step. For batch normalization, we applied *ComBat* from *sva*^109^ (v3.30.1) using a parametric adjustment for sequencing center and year of sequencing. Following batch correction, negative values were replaced with zero and the expression matrix was log_2_-transformed after adding a pseudo-count of 1. To infer immune composition in acral melanomas profiled by bulk RNA-seq (Supplementary Fig. 4), CIBERSORTx^36^ (v1.0.41) was applied with the LM22 signature matrix^110^ using B-mode batch correction, no quantile normalization, and absolute mode.

To delineate pathways associated with focal amplification of 22q11.21 (Fig. 2d), genes differentially expressed between 22q11.21-amplified and non-amplified acral melanomas were identified by constructing a linear model (*lm* function in R) to predict amplification status as a function of (1) gene expression (log_2_ adjusted) and (2) tumor stage (at the time of resection). The *t* value corresponding to the expression vector of each gene was used to rank-order the transcriptome. Pre-ranked gene set enrichment analysis (GSEA)^34^ was subsequently applied to the ranked-ordered transcriptome in order to assess HALLMARK pathways in MSigDB (v7.2)^111^.

Related to Fig. 2e, we curated a list of immunomodulatory genes, including immune checkpoint molecules, and analyzed their expression in both acral cohorts using hierarchical clustering applied with Pearson correlation and Ward D2.

### Gene fusion detection from RNA-seq data

To identify fusion genes, we aligned the RNA-seq reads for each sample to the GRCh38 human reference genome using HISAT2^112^ (v2.1.0). Candidate fusion transcripts in the sequencing reads were identified with STAR-Fusion (v1.2.0), employing the STAR aligner and FusionInspector annotator (v1.1.0) to identify the position of the chimeric RNA. For ease of manual review, the fusions were sub-grouped, with each fusion placed into the first group that either gene matched: (1) mitochondrial genes, (2) immunoglobulin genes, (3) protocadherin genes, (4) commonly expressed fusions using GTEx expression data, (5) fusions of neighboring/local_rearrangement genes, (6) non-annotated genes, and (7) all others i.e., the rare, non-local fusions of annotated protein-coding genes.

### Survival analysis

Cox proportional hazards regression (coxph in R *survival* package^113^ v3.2-7) was applied to estimate overall survival associations. Cases with an initial diagnosis preceding the sequenced tumor by more than 5 years were excluded from analysis (*n* = 5). To estimate stage- and/or TIL-adjusted associations with overall survival, stage and/or CIBERSORTx-inferred TIL content were included as a covariate. Kaplan–Meier plots for comparison of survival curves were generated either by the *survminer*^114^ package in R (v0.4.5) or by Graphpad Prism (v8).

To determine survival associations of focal CNVs identified by GISTIC (Fig. 2a, Supplementary Fig. 3a, and Supplementary Data 7), we applied Cox regression separately to each region with at least 10% recurrence frequency in either acral melanoma cohort using the copy number matrix described above (see Copy number analysis). In all cases, we dichotomized each CNV by analyzing amplified (>0) versus non-amplified (≤0) and deleted (<0) versus non-deleted (≥0). Survival *z*-scores were combined across cohorts using Stouffer’s method^115^, yielding an unweighted meta-*z*-score for each gene.

To analyze SNV- and indel-related survival associations in acral melanomas, we examined genes harboring one or more nonsynonymous mutations with at least 5% recurrence frequency in either acral melanoma cohort. These data were used to create a binary matrix in which recurrently mutated genes were rows and patients were columns (1, at least one recurrent SNV or indel; 0, otherwise). Survival *z*-scores were combined across cohorts as indicated above. The following three genes were insufficiently recurrent in at least one cohort to run Cox regression: *NF1*, *TP53*, and *ARID2*. To calculate survival associations for these genes, we randomly up-sampled patients from the Yale cohort in order to match the size of the CSU cohort. We then generated a cross-cohort survival *Z*-score for each gene (Supplementary Data 7).

To relate *LZTR1* expression to overall survival, we dichotomized patients in each cohort by determining an expression threshold that discriminates 22q11.21-amplified from non-amplified patients at a defined specificity. This was done to link the threshold for dichotomization with 22q11.21 amplification without being confounded by the upper range of *LZTR1* expression in non-amplified tumors. We used a specificity cutpoint of 95% for acral melanomas profiled in this study and sun-exposed melanomas profiled by TCGA. A specificity cutpoint of 90% was used for sun-exposed melanomas profiled in this work owing to a lack of evaluable samples in the “high” group (*n* = 1) at a specificity cutpoint of 95%. Notably, *LZTR1* expression was also significantly associated with adverse outcomes when assessed as a continuous variable (i.e., without dichotomization) in sun-exposed melanomas profiled by TCGA (*Z* = 2.86, *P* = 0.004). Skin cutaneous melanoma (SKCM) expression, copy number, and survival data from TCGA were downloaded from cBioPortal^85^.

### Single-cell RNA sequencing analysis

Single-cell RNA-seq reads from four acral melanoma patients were mapped to the GRCh38 human reference assembly and barcode-deduplicated using Cell Ranger (v3.0.2 or v5.0.1). The resulting data were imported into Seurat^116^ (v4.0.4) and quality-control filtered for the number of expressed genes per cell and the fraction of reads mapped to mitochondrial genes (Supplementary Table 5). Filtered cells were processed with Seurat using default settings for data normalization, variable feature identification, dimension reduction (PCA with 30 principal components and UMAP), and clustering. Clusters were annotated as tumor (melanoma cells) or TME using canonical lineage markers (melanoma cells: *PMEL*, *TYR*, *MITF*, and/or *MLANA*; TME: *PTPRC*, *CD3D*, *CD8A/B*, *CD19*, *CD14*, and/or *CD68* for immune cells and *PECAM1*, *FAP*, and/or *COL1A1/2* for non-immune stromal cells).

To estimate copy number alterations from scRNA-seq data, we used inferCNV (Trinity CTAT Project: https://github.com/broadinstitute/inferCNV, v1.6.0) as implemented in the R package. Per the authors’ recommendations, raw read counts of quality-controlled tumor and TME cells for each patient were provided using recommended parameters (cutoff = 0.1, cluster_by_groups = TRUE, denoise = TRUE, HMM = TRUE). Gene coordinates were obtained from GRCh37.

To characterize the RNA expression profile associated with 22q11.21 focal amplification, the log_2_-adjusted scRNA-seq dataset normalized by Census^117^ was compared between cells from a 22q11.21-amplified patient and from three non-amplified patients to identify differentially expressed genes. We used a two-sided, unpaired *t*-test with unequal variance, and the resulting *t*-statistics were used for ranking the gene list. This gene list was submitted to pre-ranked GSEA^34^ to interrogate 50 HALLMARK pathways (1000 permutations, weighted enrichment statistics, MSigDB v7.4^111^).

To predict the relative differentiation status of each melanoma cell profiled by scRNA-seq, we used integrative CytoTRACE^43^ (v0.3.3), a computational framework for inferring developmental potential on the basis of transcriptional diversity. All acral melanoma cells from YUJASMIN, YUGRUS, YUMASK, and YUPARK received a CytoTRACE score between 1 (less differentiated) and 0 (more differentiated). We verified CytoTRACE predictions using SCENT^44^ (v1.0.2) with protein–protein interaction network *net17Jan16*, and by evaluating the geometric mean of pluripotency genes^45^ (MUELLER_PLURINET, MSigDB) in melanoma cells stratified by the patient.

### Short hairpin RNA (shRNA), CRISPR-Cas9 sgRNA, and cell viability tests

We used puromycin-bearing MISSION lentiviral vectors pLKO.1 shRNA to test the effect of downregulation of target proteins on cell proliferation and signal transduction, employing scramble vector SH002 as a negative control (MISSION, Sigma-Aldrich, Supplementary Data 8), or scrambled RNA (Supplementary Data 8). LentiCRISPRv2 plasmid was obtained from Addgene (addgene.org). Guide sequences targeting *LZTR1* (Supplementary Data 8) were designed using CHOPCHOP (v3) (https://chopchop.cbu.uib.no/) and cloned into LentiCRISPRv2 to generate single sgRNA carrying plasmids following a standard method^118^. A nontarget sequence was included as the control (Supplementary Data 8).

The plasmids were packaged in lentiviral vectors with ViraPower™ Lentiviral Packaging Mix kit (Thermo Fisher, catalog no. K497500), and transfected into 293 T cells. The medium was collected and filtered with Millex-GV 33 mm PVDF filter (Millipore SLGV033RS) and then concentrated with Amicon Ultra-15 centrifugal filters (Millipore UFC910024). Melanoma cells and normal human melanocytes were infected with the lentiviruses, the medium was changed the following day, and the cells were then incubated with puromycin (2.5 µg/ml) for 5 days. Cells were collected and processed for western blotting with antibodies to target proteins (Supplementary Data 9). In addition, two days after infection the shRNA-treated cells were seeded in 96-well plates in triplicate and tested for cell viability in the absence and presence of puromycin for 72 h with the CellTiter-Glo^®^ Luminescent Cell Viability Assay, for apoptosis or RAS activity GTPase assay.

Alternatively, GV298-U6-MCS-Ubiquitin-Cherry-IRES-puromycin lentiviral plasmids were purchased from GeneChem, China. The plasmids were co-transfected with packaging plasmids (pspAX2 and pMD2G) into 293 T cells using Turbofect (Thermo Scientific) according to the manufacturer’s instructions. Lentiviruses were collected after 48 and 72 h and used to infect acral melanoma cells. Infected cells were incubated in a medium supplemented with puromycin (1 μg/ml), for 2 or 3 days, seeded in 96-well plates (2 × 10^3^/well, five replicates), and cell viability was measured with Cell Counting Kit-8 (CCK-8) (Bimake.com, China). The CCK-8 test was repeated every 24 h for 3 days.

### CRKL and LZTR1 lentivirus vectors

pDONR223-CRKL and pDONR223-LZTR1 were purchased from Addgene and DNASU, respectively. *LZTR1* was transferred into pInducer20 vector^119^ (a gift from Dr. Thomas F. Westbrook, Baylor College of Medicine), and *CRKL* to PLX304 with the Gateway LR Clonase II Enzyme mix (Thermo Fisher 11791020). The identity of each vector was validated by targeted sequencing. Lentiviruses were produced as described above and the pInducer20 *LZTR1* infected melanocytes were selected with 250 mg/ml Geneticin (G418) from American Bio (Canton, MA), catalog no. AB05057-05000, and PLX304-CRKL with 2.5 µg/ml Blasticidin S HCl from Thermo Fisher Scientific (Waltham, MA), catalog no. A1113903.

### Cell proliferation and apoptosis

The melanoma cells were grown in OptiMEM (Invitrogen, Carlsbad, CA) supplemented with 5% fetal calf serum and antibiotics. Normal human melanocytes (NBMEL) were grown from newborn foreskins in a medium supplemented with bFGF, heparin, IBMX, and dbcAMP^74^. Mouse melanocytes were grown from 1-day old newborn pups in the presence of horse serum, TPA, melanotropin, isobutyl methylxanthine, and placental extract. They became immortalized and were shifted to a medium containing only TPA after ~20 passages in cultures^120^. Some of the Yale melanoma cell lines were characterized by next-generation sequencing before^2,4^ (Supplementary Table 7).

Cell proliferation was measured with the CellTiter-Glo^®^ Luminescent Cell Viability Assay (Promega Corporation, Madison, WI). Melanoma cells were seeded in 96-well plates in triplicate or quadruplet wells after knockdown with hairpin lentivirus shRNA as indicated. Standard Error (SE) was calculated employing GraphPad Prism 7 software^121^. In addition, we seeded cells in 12-well plates (10–15,000/well) and measured proliferation by counting the number of cells from triplicate wells over a period up to 7–9 days with Beckman Cell Counter. For cell count by crystal violet, we seeded cells (3 × 10^3^/well) in six-well plates and then incubated for 10 days. Following incubation, cells were immobilized with 4% paraformaldehyde in PBS for 15 min, stained with crystal violet for 10 min, and then washed with PBS. A minimum of three random fields at 40× magnification were counted to determine cell numbers. Each sample had three replicates.

The rate of apoptosis was measured using the Caspase-Glo^®^ 3/7 Assay system (Promega catalog no. G8091) in a 96-well plate format following the manufacturer’s protocol.

PLX4032 (500 nM, Plexxikon)^121^ or LY3009120 (100 nM, Selleck, Pittsburgh, PA, catalog no. S7842) were added to the growth medium 4 h before harvesting the cells for Western blotting.

For 3D cultures, melanocytes were suspended in a 1 ml medium and seeded on 0.5% collagen (Cultrex, R&D Systems, Minneapolis, MN, catalog no. 3442-050-01), in 24 well plates for 3 days.

### Microscopy

Images were acquired using an inverted Nikon Eclipse Ti fluorescence microscope with a Plan Apochromat lambda 60X /1.40 Oil objective or a Plan Fluor 4X/0.13 objective for fluorescent images or DIC images, respectively, a CSU-W1 confocal spinning disk unit, an iXon Ultra 888 camera (Andor Technology), MLC 400B laser unit (Agilent Technologies), and NIS Elements software (Nikon).

### Western blotting and antibodies

We used western blots to identify the levels of proteins as previously described^67^. Cell extracts (20 µg/lane) were fractionated in 3–8% or 4–12% tris-acetate gel, transferred to membranes (NuPAGE Life Technologies, catalog no. NP0006) that were probed with the primary antibodies listed in Supplementary Data 9. All antibodies were used at the concentrations recommended by the manufacturers (Supplementary Data 9).

### RAS activity assay

The amount of GTP-bound RAS was determined using the Ras-GTPase Chemi ELISA Kit (Active Motif, Carlsbad, CA) following the manufacturer’s protocol. Melanoma cells treated with control shRNA or *shLZTR1* were collected 5 days after infection by scraping on ice, washed with cold PBS, lysed, centrifuged and 50 µg protein/assay, in triplicates, were used following the manufacturer instructions.

### Immunostaining

Cells were grown on the surface of four-well slides, washed two to three times with PBS, fixed with 4% paraformaldehyde for 15 min at room temperature, washed three times with PBS, permeabilized with 0.2% NP40 in PBS for 5 min, washed with PBS and incubated in PBS containing 1% BSA or (blocking buffer) for 1 h. The cells were incubated with anti-GM130 antibody (clone 4A3 Millipore, Mouse), or calnexin (mouse mAb) for 1 h at room temperature, and stained with secondary Alexa Fluor (Cy2) diluted in blocking buffer 1:1,000 for 1 h (Supplementary Data 9). They were washed 3X with PBS, incubated with rhodamine-phalloidin to stain actin and DAPI to stain the nucleus.

### Statistical analysis

Linear relationships were modeled by linear regression (*R*^2^), and a *t*-test was used to assess whether the result was significantly nonzero. When data were normally distributed, group comparisons were determined using a two-sided *t*-test with unequal variance or a paired *t*-test, as appropriate; otherwise, a two-sided Wilcoxon test was applied. Results with *P* < 0.05 were considered significant. Data analyses were performed with R (v3.5 + ), Python (v3.8.5), and Prism (v7 or v8, GraphPad Software, Inc.). The investigators were not blinded to allocation during experiments and outcome assessment. No sample-size estimates were performed to ensure adequate power to detect a prespecified effect size.

### Reporting summary

Further information on research design is available in the Nature Research Reporting Summary linked to this article.

## Supplementary information

Supplementary Information

Description of Additional Supplementary Files

Supplementary Data 1

Supplementary Data 2

Supplementary Data 3

Supplementary Data 4

Supplementary Data 5

Supplementary Data 6

Supplementary Data 7

Supplementary Data 8

Supplementary Data 9

Reporting Summary

## Data Availability

The Liang et al. publicly available WES data^11^ used in this study are available in the dbGAP database under accession code phs001036.v1.p1. All raw DNA sequencing data and raw bulk/single-cell RNA sequencing data generated in this study from the Yale cohort have been deposited in the dbGAP database under accession code phs000933.v3.p1. The data are available under restricted access, access can be obtained by submitting a project request to dbGAP (https://dbgap.ncbi.nlm.nih.gov/aa/wga.cgi). All raw DNA and RNA sequencing data generated in this study from the CSU cohort have been deposited in the Genome Sequence Archive (GSA) database under accession code HRA001648. The data are available under restricted access, access can be obtained by submitting a request to the corresponding Data Access Committee (Contact person: Peng Cong, Email: pengcongxy@csu.edu.cn). The processed bulk RNA sequencing data (Yale and CSU cohorts) and processed single-cell RNA sequencing data (Yale cohort) have been deposited in the Gene Expression Omnibus (GEO) database under accession code GSE162682. The remaining data are available within the Article, Supplementary Information, or Supplementary Data files. Source data are provided with this paper.

## Source data

Source Data

## Code availability

Custom scripts used in this study are publicly available from https://github.com/knightjimr/CNVL.

## References

[1] Curtin JA (2005). Distinct sets of genetic alterations in melanoma. N. Engl. J. Med..

[2] Krauthammer M (2012). Exome sequencing identifies recurrent somatic RAC1 mutations in melanoma. Nat. Genet..

[3] Hodis E (2012). A landscape of driver mutations in melanoma. Cell.

[4] Krauthammer M (2015). Exome sequencing identifies recurrent mutations in NF1 and RASopathy genes in sun-exposed melanomas. Nat. Genet.

[5] Network TCGA (2015). Genomic classification of cutaneous melanoma. Cell.

[6] Zhang T, Dutton-Regester K, Brown KM, Hayward NK (2016). The genomic landscape of cutaneous melanoma. Pigment Cell Melanoma Res..

[7] Bastian BC (2000). Gene amplifications characterize acral melanoma and permit the detection of occult tumor cells in the surrounding skin. Cancer Res..

[8] Bastian BC, Olshen AB, LeBoit PE, Pinkel D (2003). Classifying melanocytic tumors based on DNA copy number changes. Am. J. Pathol..

[9] Furney SJ (2012). Genomic characterisation of acral melanoma cell lines. Pigment Cell Melanoma Res..

[10] Kong Y (2016). Analysis of mTOR gene aberrations in melanoma patients and evaluation of their sensitivity to PI3K-AKT-mTOR pathway inhibitors. Clin. Cancer Res..

[11] Liang WS (2017). Integrated genomic analyses reveal frequent TERT aberrations in acral melanoma. Genome Res..

[12] Kong Y (2017). Frequent genetic aberrations in the CDK4 pathway in acral melanoma indicate the potential for CDK4/6 inhibitors in targeted therapy. Clin. Cancer Res..

[13] Hayward NK (2017). Whole-genome landscapes of major melanoma subtypes. Nature.

[14] Yeh I (2019). Targeted genomic profiling of acral melanoma. J. Natl Cancer Inst..

[15] Newell F (2020). Whole-genome sequencing of acral melanoma reveals genomic complexity and diversity. Nat. Commun..

[16] Park CK, Kim SK (2017). Clinicopathological significance of intratumoral and peritumoral lymphocytes and lymphocyte score based on the histologic subtypes of cutaneous melanoma. Oncotarget.

[17] Klemen ND (2020). Survival after checkpoint inhibitors for metastatic acral, mucosal and uveal melanoma. J. Immunother. Cancer.

[18] Curtin JA, Busam K, Pinkel D, Bastian BC (2006). Somatic activation of KIT in distinct subtypes of melanoma. J. Clin. Oncol..

[19] Beadling C (2008). KIT gene mutations and copy number in melanoma subtypes. Clin. Cancer Res..

[20] Vazquez Vde L (2016). Molecular profiling, including TERT promoter mutations, of acral lentiginous melanomas. Melanoma Res..

[21] Shim JH (2017). Mutational profiling of acral melanomas in Korean populations. Exp. Dermatol.

[22] Moon KR (2018). Genetic alterations in primary acral melanoma and acral melanocytic nevus in Korea: common mutated genes show distinct cytomorphological features. J. Invest. Dermatol..

[23] Zaremba A (2019). Clinical and genetic analysis of melanomas arising in acral sites. Eur. J. Cancer.

[24] Puig-Butille JA (2013). Genetic alterations in RAS-regulated pathway in acral lentiginous melanoma. Exp. Dermatol..

[25] Yu S (2018). TERT copy gain predicts the outcome of high-dose interferon alpha-2b therapy in acral melanoma. Onco Targets Ther..

[26] Furney SJ (2014). The mutational burden of acral melanoma revealed by whole-genome sequencing and comparative analysis. Pigment Cell Melanoma Res..

[27] Shi, K. et al. Distinct genomic features in a retrospective cohort of mucosal, acral and vulvovaginal melanomas. *J. Am. Acad. Dermatol*. 10.1016/j.jaad.2019.07.017 (2019). 10.1016/j.jaad.2019.07.017 31306728

[28] Behbahani, S., Malerba, S. & Samie, F. H. Racial and ethnic differences in the clinical presentation and outcomes of acral lentiginous melanoma. *Br. J. Dermatol.* **184**, 158–160 (2021). 10.1111/bjd.19406 32683697

[29] Bradford PT, Goldstein AM, McMaster ML, Tucker MA (2009). Acral lentiginous melanoma: incidence and survival patterns in the United States, 1986-2005. Arch. Dermatol.

[30] Smit NP (2008). Increased melanogenesis is a risk factor for oxidative DNA damage–study on cultured melanocytes and atypical nevus cells. Photochem. Photobio..

[31] Walker GJ (1998). Virtually 100% of melanoma cell lines harbor alterations at the DNA level within CDKN2A, CDKN2B, or one of their downstream targets. Genes Chromosomes Cancer.

[32] Jönsson G (2007). Genomic profiling of malignant melanoma using tiling-resolution arrayCGH. Oncogene.

[33] Mermel CH (2011). GISTIC2.0 facilitates sensitive and confident localization of the targets of focal somatic copy-number alteration in human cancers. Genome Biol..

[34] Subramanian A (2005). Gene set enrichment analysis: a knowledge-based approach for interpreting genome-wide expression profiles. Proc. Natl Acad. Sci. USA.

[35] Madden E, Logue SE, Healy SJ, Manie S, Samali A (2019). The role of the unfolded protein response in cancer progression: From oncogenesis to chemoresistance. Biol. Cell.

[36] Newman AM (2019). Determining cell type abundance and expression from bulk tissues with digital cytometry. Nat. Biotechnol..

[37] Baumjohann D, Brossart P (2021). T follicular helper cells: linking cancer immunotherapy and immune-related adverse events. J. Immunother. Cancer.

[38] Intlekofer AM, Finley LWS (2019). Metabolic signatures of cancer cells and stem cells. Nat. Metab..

[39] Schatton T (2008). Identification of cells initiating human melanomas. Nature.

[40] Abbaszadegan MR (2017). Isolation, identification, and characterization of cancer stem cells: A review. J. Cell Physiol..

[41] Eun K, Ham SW, Kim H (2017). Cancer stem cell heterogeneity: origin and new perspectives on CSC targeting. BMB Rep..

[42] Thankamony AP, Saxena K, Murali R, Jolly MK, Nair R (2020). Cancer stem cell plasticity - a deadly deal. Front. Mol. Biosci..

[43] Gulati GS (2020). Single-cell transcriptional diversity is a hallmark of developmental potential. Science.

[44] Teschendorff AE, Enver T (2017). Single-cell entropy for accurate estimation of differentiation potency from a cell’s transcriptome. Nat. Commun..

[45] Müller F-J (2008). Regulatory networks define phenotypic classes of human stem cell lines. Nature.

[46] Frattini V (2013). The integrated landscape of driver genomic alterations in glioblastoma. Nat. Genet.

[47] Chen RH (2020). Cullin 3 and its role in tumorigenesis. Adv. Exp. Med. Biol..

[48] Pathmanaban ON (2017). Association of genetic predisposition with solitary schwannoma or meningioma in children and young adults. JAMA Neurol..

[49] Evans DG (2018). Schwannomatosis: a genetic and epidemiological study. J. Neurol. Neurosurg. Psychiatry.

[50] Luo LY, Hahn WC (2015). Oncogenic signaling adaptor proteins. J. Genet. Genomics.

[51] Cheung HW (2011). Amplification of CRKL induces transformation and epidermal growth factor receptor inhibitor resistance in human non-small cell lung cancers. Cancer Disco..

[52] Kim YH (2010). Genomic and functional analysis identifies CRKL as an oncogene amplified in lung cancer. Oncogene.

[53] Kostrzewska-Poczekaj M (2020). Copy number gains of the putative CRKL oncogene in laryngeal squamous cell carcinoma result in strong nuclear expression of the protein and influence cell proliferation and migration. Sci. Rep..

[54] Bigenzahn JW (2018). LZTR1 is a regulator of RAS ubiquitination and signaling. Science.

[55] Steklov M (2018). Mutations in LZTR1 drive human disease by dysregulating RAS ubiquitination. Science.

[56] Umeki I (2019). Delineation of LZTR1 mutation-positive patients with Noonan syndrome and identification of LZTR1 binding to RAF1-PPP1CB complexes. Hum. Genet.

[57] Motta M (2019). Dominant Noonan syndrome-causing LZTR1 mutations specifically affect the Kelch domain substrate-recognition surface and enhance RAS-MAPK signaling. Hum. Mol. Genet..

[58] Li X (2019). Molecular and phenotypic spectrum of Noonan syndrome in Chinese patients. Clin. Genet.

[59] Guemes, M. et al. LZTR1: genotype expansion in Noonan syndrome. *Horm. Res. Paediatr.* **92**, 269–275 (2019). 10.1159/000502741 31533111

[60] Abe T (2020). LZTR1 facilitates polyubiquitination and degradation of RAS-GTPases. Cell Death Differ..

[61] Julien O, Wells JA (2017). Caspases and their substrates. Cell Death Differ..

[62] Nacak TG, Leptien K, Fellner D, Augustin HG, Kroll J (2006). The BTB-kelch protein LZTR-1 is a novel Golgi protein that is degraded upon induction of apoptosis. J. Biol. Chem..

[63] Mukherjee S, Chiu R, Leung SM, Shields D (2007). Fragmentation of the Golgi apparatus: an early apoptotic event independent of the cytoskeleton. Traffic.

[64] Fisher DE (2000). Microphthalmia: a signal responsive transcriptional regulator in development. Pigment Cell Res..

[65] Wu M (2000). c-Kit triggers dual phosphorylations, which couple activation and degradation of the essential melanocyte factor Mi. Genes Dev..

[66] Phung B (2017). KITD816V induces SRC-mediated tyrosine phosphorylation of MITF and altered transcription program in melanoma. Mol. Cancer Res..

[67] Halaban R (2019). A novel anti-melanoma SRC-family kinase inhibitor. Oncotarget.

[68] Lin F (2015). CRKL promotes lung cancer cell invasion through ERK-MMP9 pathway. Mol. Carcinog..

[69] Franke, F. C. et al. Novel role for CRK adaptor proteins as essential components of SRC/FAK signaling for epithelial-mesenchymal transition and colorectal cancer aggressiveness. *Int. J. Cancer* **147**, 1715–1731 (2020). 10.1002/ijc.32955 32147820

[70] Bates RC, Edwards NS, Yates JD (2000). Spheroids and cell survival. Crit. Rev. Oncol. Hematol..

[71] Haass NK, Smalley KS, Li L, Herlyn M (2005). Adhesion, migration and communication in melanocytes and melanoma. Pigment Cell Res..

[72] Chaudhuri O, Cooper-White J, Janmey PA, Mooney DJ, Shenoy VB (2020). Effects of extracellular matrix viscoelasticity on cellular behaviour. Nature.

[73] Arozarena I, Wellbrock C (2019). Phenotype plasticity as enabler of melanoma progression and therapy resistance. Nat. Rev. Cancer.

[74] Halaban, R. in *Culture of Human Tumor Cells* (eds Freshney, R. I. & Pfranger, R.) Ch. 12 (John Wiley & Sons, Inc., 2004).

[75] Halaban R, Cheng E, Zhang Y, Mandigo CE, Miglarese MR (1998). Release of cell cycle constraints in mouse melanocytes by overexpressed mutant E2F1_E132_, but not by deletion of p16^INK4A^ or p21^WAF1/CIP1^. Oncogene.

[76] Wang Y, Zhao Y, Ma S (2016). Racial differences in six major subtypes of melanoma: descriptive epidemiology. BMC Cancer.

[77] Lv J, Dai B, Kong Y, Shen X, Kong J (2016). Acral melanoma in Chinese: a clinicopathological and prognostic study of 142 cases. Sci. Rep..

[78] De Wet J, Tod B, Visser WI, Jordaan HF, Schneider JW (2018). Clinical and pathological features of acral melanoma in a South African population: a retrospective study. S. Afr. Med. J..

[79] Wada M (2017). Acral lentiginous melanoma versus other melanoma: a single-center analysis in Japan. J. Dermatol..

[80] Sheen YS (2017). A clinicopathological analysis of 153 acral melanomas and the relevance of mechanical stress. Sci. Rep..

[81] Desai A, Ugorji R, Khachemoune A (2017). Acral melanoma foot lesions. Part 1: epidemiology, aetiology, and molecular pathology. Clin. Exp. Dermatol..

[82] Madankumar R (2016). Acral melanocytic lesions in the United States: prevalence, awareness, and dermoscopic patterns in skin-of-color and non-Hispanic white patients. J. Am. Acad. Dermatol.

[83] Johnston JJ (2018). Autosomal recessive Noonan syndrome associated with biallelic LZTR1 variants. Genet. Med..

[84] Dhanoa BS, Cogliati T, Satish AG, Bruford EA, Friedman JS (2013). Update on the Kelch-like (KLHL) gene family. Hum. Genomics.

[85] Cerami E (2012). The cBio cancer genomics portal: an open platform for exploring multidimensional cancer genomics data. Cancer Disco..

[86] Gao J (2013). Integrative analysis of complex cancer genomics and clinical profiles using the cBioPortal. Sci. Signal.

[87] McGary EC, Lev DC, Bar-Eli M (2002). Cellular adhesion pathways and metastatic potential of human melanoma. Cancer Biol. Ther..

[88] Rathinam R, Berrier A, Alahari SK (2011). Role of Rho GTPases and their regulators in cancer progression. Front. Biosci..

[89] Ridley AJ (2003). Cell migration: integrating signals from front to back. Science.

[90] Li H, Durbin R (2009). Fast and accurate short read alignment with Burrows-Wheeler transform. Bioinformatics.

[91] McKenna A (2010). The genome analysis toolkit: a MapReduce framework for analyzing next-generation DNA sequencing data. Genome Res..

[92] Wang K, Li M, Hakonarson H (2010). ANNOVAR: functional annotation of genetic variants from high-throughput sequencing data. Nucleic Acids Res..

[93] McLaren W (2016). The ensembl variant effect predictor. Genome Biol..

[94] Lek M (2016). Analysis of protein-coding genetic variation in 60,706 humans. Nature.

[95] Zhao S (2013). Landscape of somatic single-nucleotide and copy-number mutations in uterine serous carcinoma. Proc. Natl Acad. Sci. USA.

[96] Cocco E (2016). Dual CCNE1/PIK3CA targeting is synergistic in CCNE1-amplified/PIK3CA-mutated uterine serous carcinomas in vitro and in vivo. Br. J. Cancer.

[97] Bi M (2016). Genomic characterization of sarcomatoid transformation in clear cell renal cell carcinoma. Proc. Natl Acad. Sci. USA.

[98] Zhao S (2016). Mutational landscape of uterine and ovarian carcinosarcomas implicates histone genes in epithelial–mesenchymal transition. Proc. Natl Acad. Sci. USA.

[99] Choi J (2021). Integrated mutational landscape analysis of uterine leiomyosarcomas. Proc. Natl Acad. Sci. USA.

[100] Zhao S (2016). Mutational landscape of uterine and ovarian carcinosarcomas implicates histone genes in epithelial-mesenchymal transition. Proc. Natl Acad. Sci. USA.

[101] Carter SL (2012). Absolute quantification of somatic DNA alterations in human cancer. Nat. Biotechnol..

[102] Lawrence MS (2013). Mutational heterogeneity in cancer and the search for new cancer-associated genes. Nature.

[103] Gu Z, Eils R, Schlesner M (2016). Complex heatmaps reveal patterns and correlations in multidimensional genomic data. Bioinformatics.

[104] Patro R, Duggal G, Love MI, Irizarry RA, Kingsford C (2017). Salmon provides fast and bias-aware quantification of transcript expression. Nat. Methods.

[105] Frankish A (2019). GENCODE reference annotation for the human and mouse genomes. Nucleic Acids Res..

[106] Soneson C, Love MI, Robinson MD (2015). Differential analyses for RNA-seq: transcript-level estimates improve gene-level inferences. F1000Res.

[107] Cunningham F (2018). Ensembl 2019. Nucleic Acids Res..

[108] Durinck S, Spellman PT, Birney E, Huber W (2009). Mapping identifiers for the integration of genomic datasets with the R/Bioconductor package biomaRt. Nat. Protoc..

[109] Leek JT, Johnson WE, Parker HS, Jaffe AE, Storey JD (2012). The sva package for removing batch effects and other unwanted variation in high-throughput experiments. Bioinformatics.

[110] Newman AM (2015). Robust enumeration of cell subsets from tissue expression profiles. Nat. Methods.

[111] Liberzon A (2015). The molecular signatures database (MSigDB) hallmark gene set collection. Cell Syst..

[112] Kim D, Langmead B, Salzberg SL (2015). HISAT: a fast spliced aligner with low memory requirements. Nat. Methods.

[113] Therneau, T. M. & Grambsch, P. M. In *Modeling Survival Data: Extending the Cox Model* (eds Therneau, T. M. & Grambsch, P. M.) Ch. 10 (Springer, 2000).

[114] Alboukadel, K., Marcin, K. & Przemyslaw, B. Survminer: drawing survival curves using’ggplot2’. R package version 0.4.5. (2019).

[115] Stouffer, S. A., Suchman, E. A., Devinney, L. C., Star, S. A. & Williams Jr, R. M. *The American Soldier: Adjustment During Army Life.* (*Studies in Social Psychology in World War II*) Vol. 1 (Princeton Univ. Press, 1949).

[116] Stuart T (2019). Comprehensive integration of single-cell data. Cell.

[117] Qiu X (2017). Single-cell mRNA quantification and differential analysis with census. Nat. Methods.

[118] Ran FA (2013). Genome engineering using the CRISPR-Cas9 system. Nat. Protoc..

[119] Meerbrey KL (2011). The pINDUCER lentiviral toolkit for inducible RNA interference in vitro and in vivo. Proc. Natl Acad. Sci. USA.

[120] Tamura A (1987). Normal murine melanocytes in culture. Vitr. Cell. Dev. Biol..

[121] Halaban R (2010). PLX4032, a selective BRAF(V600E) kinase inhibitor, activates the ERK pathway and enhances cell migration and proliferation of BRAF(WT) melanoma cells. Pigment Cell Melanoma Res..

[122] Tate JG (2019). COSMIC: the catalogue of somatic mutations in cancer. Nucleic Acids Res..

[123] Sondka Z (2018). The COSMIC cancer gene census: describing genetic dysfunction across all human cancers. Nat. Rev. Cancer.

