# Author Correction: Integrative molecular and clinical profiling of acral melanoma links focal amplification of 22q11.21 to metastasis

**DOI:** 10.1038/s41467-022-30446-w

**Published:** 2022-05-10

**Authors:** Farshad Farshidfar, Kahn Rhrissorrakrai, Chaya Levovitz, Cong Peng, James Knight, Antonella Bacchiocchi, Juan Su, Mingzhu Yin, Mario Sznol, Stephan Ariyan, James Clune, Kelly Olino, Laxmi Parida, Joerg Nikolaus, Meiling Zhang, Shuang Zhao, Yan Wang, Gang Huang, Miaojian Wan, Xianan Li, Jian Cao, Qin Yan, Xiang Chen, Aaron M. Newman, Ruth Halaban

**Affiliations:** 1 Institute for Stem Cell Biology and Regenerative Medicine, Stanford University, Stanford, CA USA; 2 Department of Biomedical Data Science, Stanford University, Stanford, CA USA; 3 IBM Research, Yorktown Heights, NY USA; 4 Xiangya Hospital, Central South University, Changsha, China; 5 Yale Center for Genome Analysis, Yale University, New Haven, CT 06520 USA; 6 Department of Dermatology, Yale University School of Medicine, New Haven, CT USA; 7 Department of Pathology, Yale University School of Medicine, New Haven, CT USA; 8 Department of Internal Medicine, Section of Medical Oncology, Yale University School of Medicine, New Haven, CT USA; 9 Department of Surgery, Yale University School of Medicine, New Haven, CT USA; 10 Department of Molecular and Cellular Physiology, Yale University School of Medicine, New Haven, CT USA; 11 Department of Dermatologic Surgery Institute of Dermatology, Chinese Academy of Medical Sciences & Peking Union Medical College, Nanjing, China; 12 Department of Bone and Soft Tissue oncology, Hunan Cancer Hospital, Affiliated Tumor Hospital of Xiangya Medical School of Central South University, Changsha, Hunan China; 13 Department of Dermatology, The Third Affiliated Hospital, Sun Yat-sen University, Guangzhou, China

**Keywords:** Melanoma, Tumour biomarkers, Tumour heterogeneity, Cancer genomics, Melanoma, Tumour biomarkers, Tumour heterogeneity, Cancer genomics

Correction to: *Nature Communications* 10.1038/s41467-022-28566-4, published online 25 February 2022.

The original version of the Supplementary Information associated with this Article included an incorrect Supplementary Data 3 file, in which the ‘MAF Sun-Exposed Melanoma’ tab included only 65,536 rows instead of 122,339 rows. The HTML has been updated to include a corrected version of Supplementary Data 3.

## Supplementary information

Updated Supplementary Data 3

