# Supplementary material for: Integrative molecular and clinical profiling of acral melanoma links focal amplification of 22q11.21 to metastasis
Source: Nat Commun. 2022 Feb 23;13:898. doi: 10.1038/s41467-022-28566-4 (PMC8866401; doi:10.1038/s41467-022-28566-4)
Supplement: Supplementary file 1 — Supplementary Information [file 41467_2022_28566_MOESM1_ESM.pdf]

# Supplementary Information

## Integrative molecular and clinical profiling of acral melanoma links focal amplification of 22q11.21 to metastasis

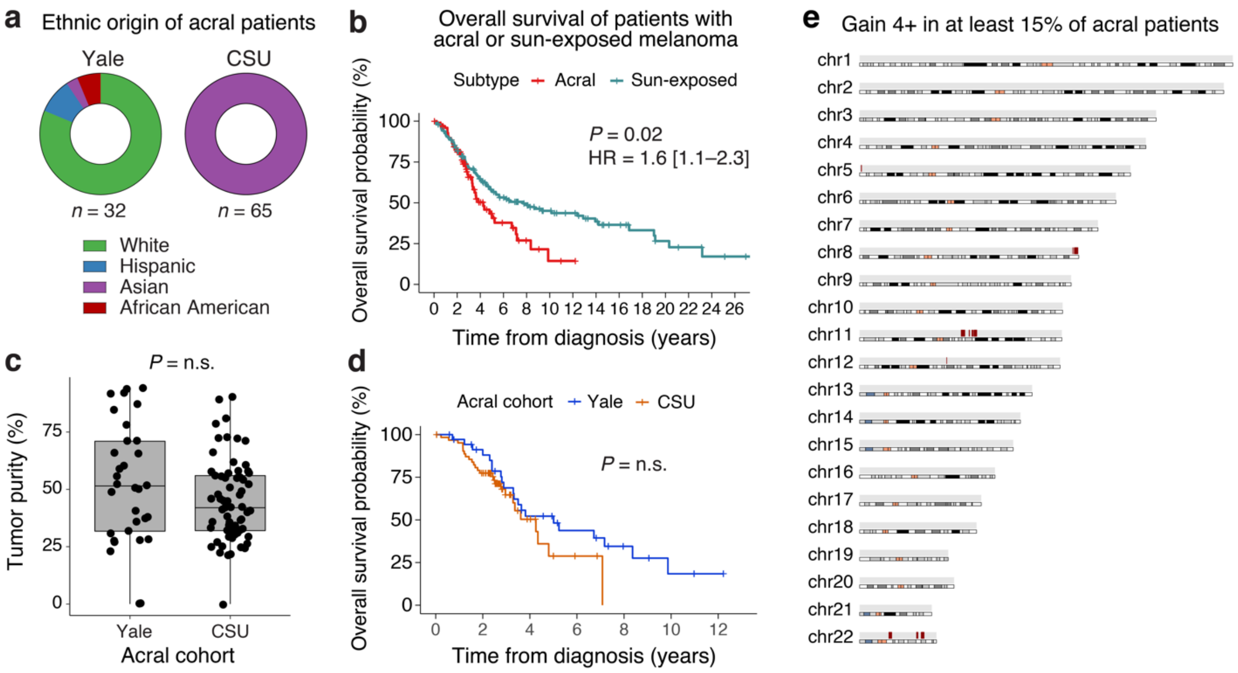


**Supplementary Figure 1: Characteristics and overall survival patterns of patients analyzed in this study. a** Pie charts depicting ethnic origin of acral melanoma patients in each cohort (Table 1). **b** Kaplan-Meier plot showing overall survival in acral versus sun-exposed melanoma subtypes analyzed in this work. Statistical significance was calculated with a two-sided log-rank test. HR, hazard ratio; HR 95% confidence interval is shown in brackets. **c** Tumor purity of acral melanoma samples profiled by whole exome sequencing in Yale (*n* = 32) and CSU (*n* = 65) cohorts (Methods). Group comparison was performed by a two-sided, unpaired Wilcoxon rank sum test. Box center lines, bounds of the box, and whiskers indicate medians, 1$^{st}$ and 3$^{rd}$ quartiles, and minimum and maximum values within 1.5 × IQR (interquartile range) of the box limits, respectively. **d** Kaplan-Meier plot showing overall survival of acral melanoma patients in Yale versus CSU cohorts. Statistical significance was calculated with a two-sided log-rank test. n.s., not significant. **e** Karyoplot of recurrent amplifications in acral melanoma. Chromosomal location of genes with a gain (red), defined as CNV ≥ 4 in ≥ 15% of acral melanomas analyzed in this work demonstrate specific patterning of concentrated amplification events. Source data are provided as a Source Data file.

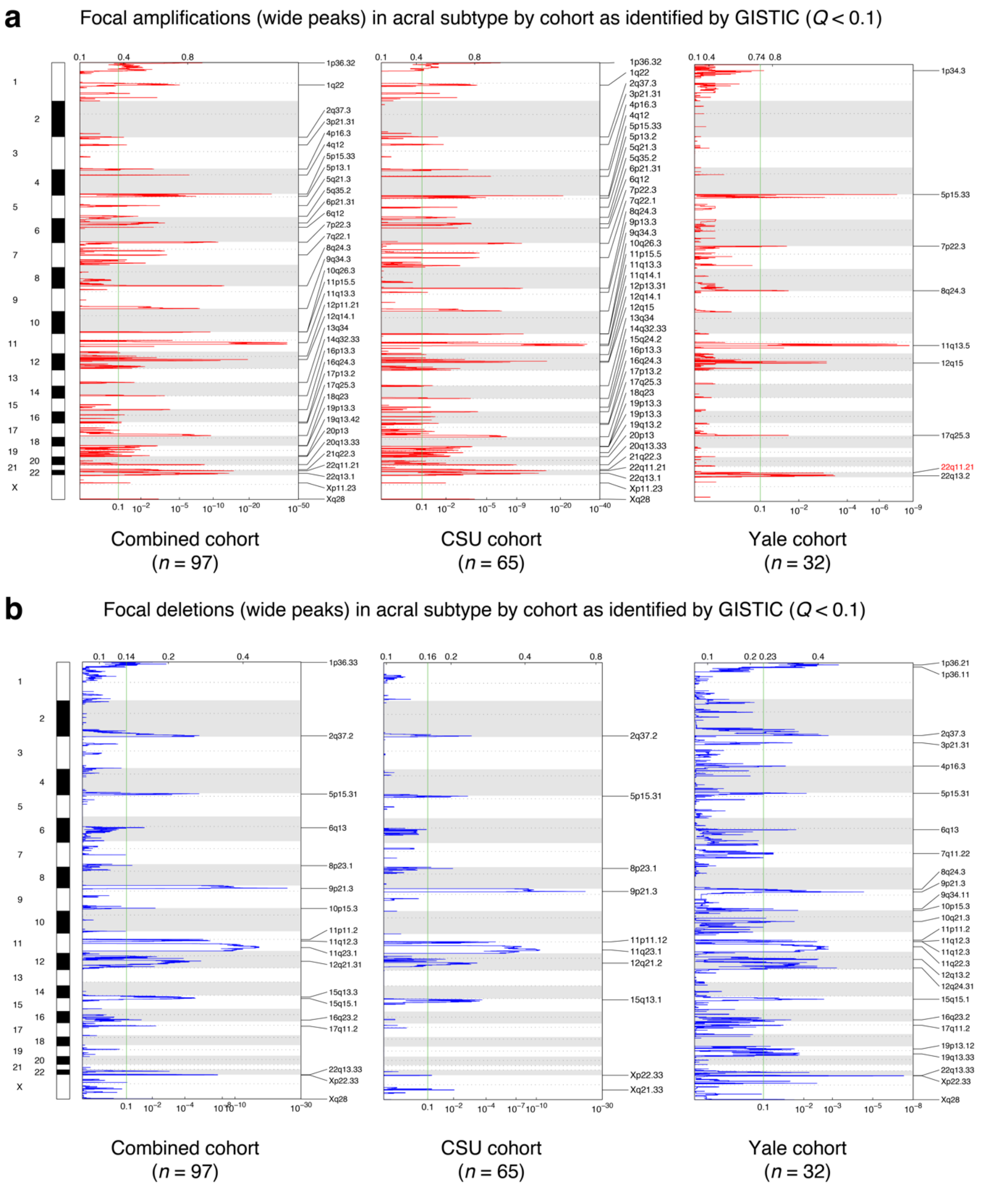


**Supplementary Figure 2: Landscape of focal amplifications and deletions in acral melanoma**. **a** Focally-amplified wide peaks in acral melanoma tumors identified by Genomic Identification of Significant Targets in Cancer (GISTIC) (*Left*, all tumors; *Center*, CSU cohort; *Right*, Yale cohort). Labeled cytobands denote wide peaks with $Q < 0.1$. Although significant, 22q11.21 was not automatically labeled by GISTIC in the Yale cohort and is therefore indicated in red text. **b** Same as a but showing focally-deleted wide peaks. Source data are provided as a Source Data file.

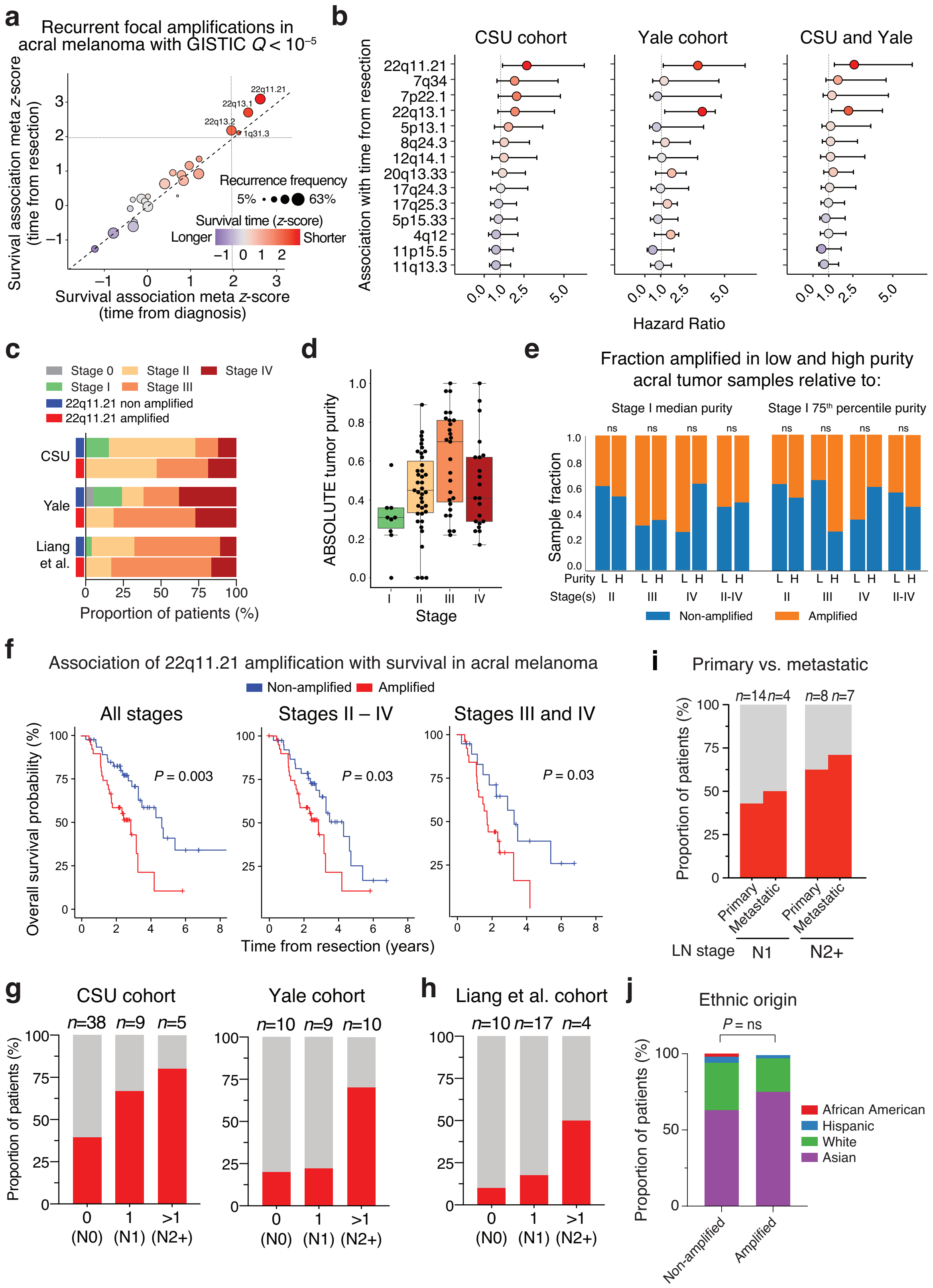


**Supplementary Figure 3: Associations of 22q11.21 amplification status with survival, stage, and lymph node status in acral melanoma. a** Same as Fig. 2a but

showing only recurrent focal amplifications identified in acral melanomas (CSU and Yale combined) with GISTIC $Q < 10^{-5}$ (Supplementary Data 4). **b** Association between significant focal amplifications defined in the CSU cohort (GISTIC $Q < 10^{-5}$; Supplementary Data 4) and overall survival (from date of tumor resection), shown as hazard ratios (circles) with 95% confidence intervals (error bars). Red indicates hazard ratio (HR) > 1 (shorter survival time) and blue indicates HR < 1 (longer survival time). Notably, while HRs are shown for CSU (patient $n$ = 60), Yale (patient $n$ = 29), and CSU + Yale (combined, $n$ = 89), focal amplifications in this analysis were defined from the CSU cohort alone. **c** Stage distribution in patients with acral melanoma, shown as a function of 22q11.21 focal amplification status in three independent cohorts (CSU $n$ = 65, Yale $n$ = 32, Liang et al. $n$ = 34). **d** Estimated tumor purity (ABSOLUTE) by stage (Stage I $n$ = 9, Stage II $n$ = 39, Stage III $n$ = 27, Stage IV $n$ = 21) in patients with acral melanoma. Box center lines, bounds of the box, and whiskers indicate medians, 1$^{st}$ and 3$^{rd}$ quartiles, and minimum and maximum values within 1.5 × IQR (interquartile range) of the box limits, respectively. **e** Stacked bar plots showing the proportions of 22q11.21-amplified vs. non-amplified acral melanomas with tumor purity estimates above or below tumor purity thresholds (median or 75$^{th}$ percentile) defined from stage I tumors using ABSOLUTE. L, tumor purity estimates are lower than the threshold; H, tumor purity estimates are higher than the threshold. Statistical significance comparing the number of 22q11 amplified tumors above and below the threshold for each stage (or range of stages) was assessed by Fisher's exact test. No significant differences were observed. ns, not significant. **f** Association of chr22q11.21 amplification with overall survival in acral melanoma (CSU and Yale cohorts), shown for all stages, stage II to IV disease, and advanced disease (stage III and IV). Statistical significance was assessed by a two-sided log-rank test. **g** Frequency of 22q11.21-amplified acral melanoma patients shown as a function of the number of positive lymph nodes. **h** Same as g but for patients from Liang et al. **i** Frequency of 22q11.21-amplilfied acral melanoma patients shown as a function of lymph node (LN) stage and stratified by tumor site of origin (primary or metastatic). **j** Ethnic distribution of acral melanoma patients shown as a function of 22q11.21 focal amplification status (amplified patients $n$ = 44, non-amplified patients $n$ = 53). Statistical significance was assessed by a Chi-square test for trend. ns, not significant. Source data are provided as a Source Data file.

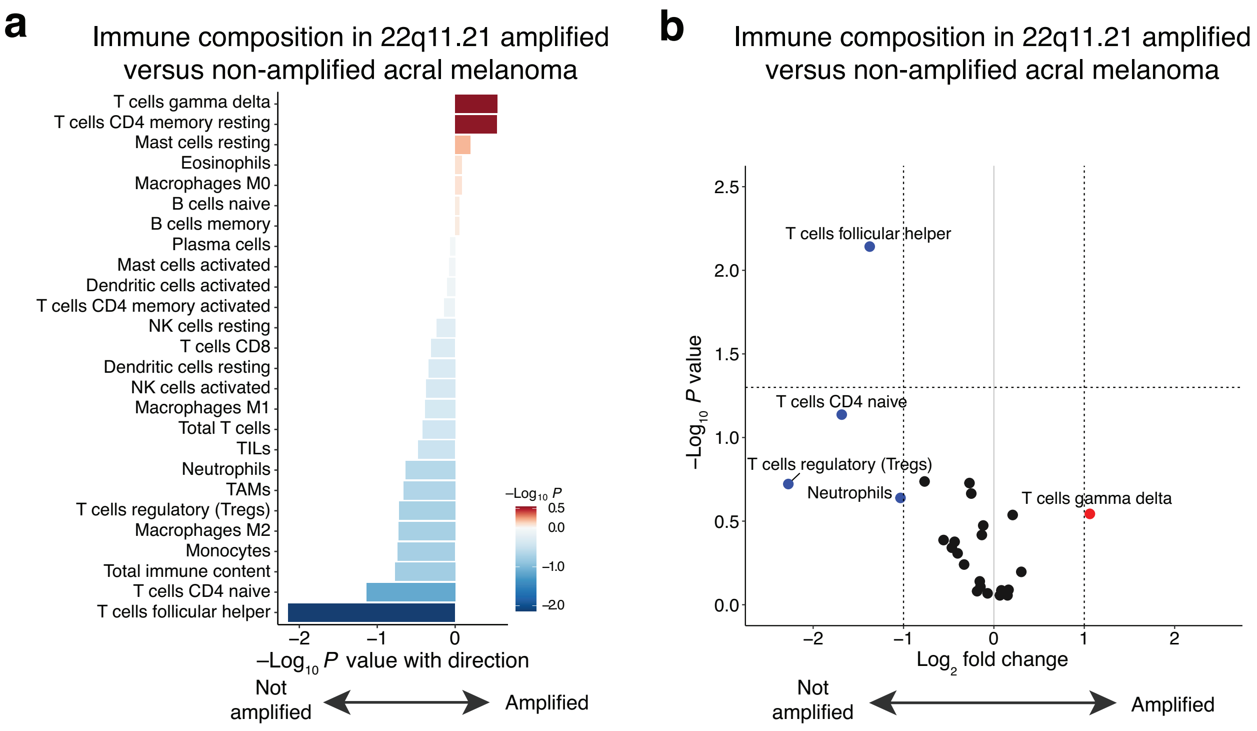


**Supplementary Figure 4: Immune composition of 22q11.21-amplified acral melanoma. a** Analysis of immune composition in 22q11.21-amplified versus non-amplified acral melanomas profiled by bulk RNA-seq (*n* = 38). The frequencies of 22 immune subsets and four broader immune phenotypes were inferred by CIBERSORTx. Statistical significance was determined by a two-sided, unpaired Wilcoxon rank sum test, expressed as $-\log_{10}$ p-values with directionality (negative and positive values denote immune subsets that are higher in non-amplified and amplified tumors, respectively). TILs, tumor-infiltrating lymphocytes; TAMs, tumor-associated myeloid cells. For details, see Methods. **b** Same as a but also showing the average $\log_2$ fold change between amplified and non-amplified acral melanomas. Source data are provided as a Source Data file.

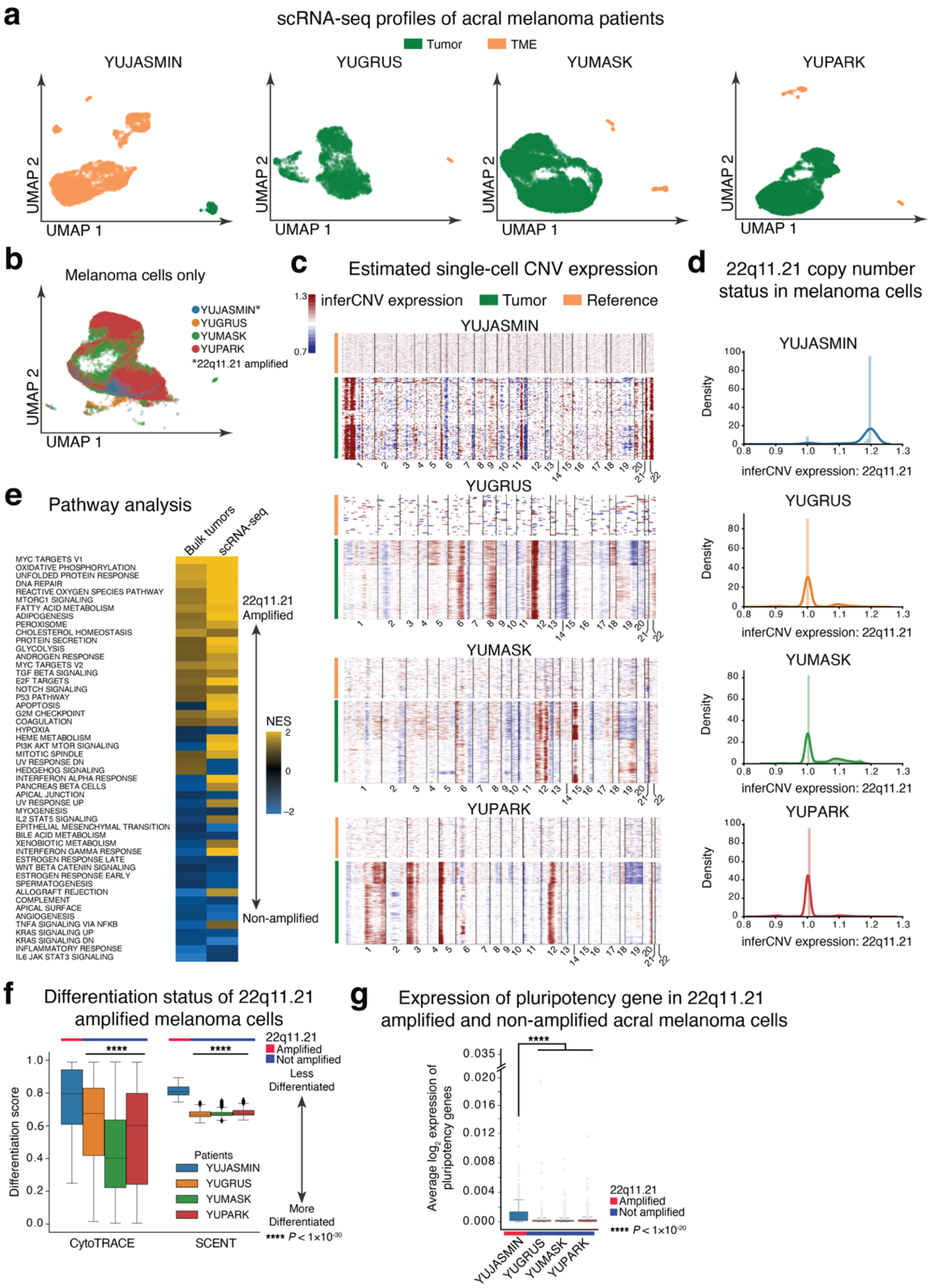


**Supplementary Figure 5: Single-cell transcriptomic analysis of 22q11.21-amplified and non-amplified acral melanoma. a** UMAP projections of four acral

melanoma specimens profiled by scRNA-seq, distinguishing melanoma cells ('Tumor') from immune and stromal lineages ('TME') (Supplementary Data 1). **b** Same as a but restricted to melanoma cells after integration across patients. **c** Heat maps depicting the results of applying single-cell copy number inference (inferCNV) to four acral melanoma samples, where TME cells in panel a were used as a normal reference, paired for each patient. **d** Density plots showing the mean expression of 22q11.21 genes in each patient following inferCNV normalization (related to panel c). Only YUJASMIN is 22q11.21-amplified as determined by WES and scRNA-seq-informed copy number inference. **e** Pre-ranked gene set enrichment analysis (GSEA) showing concordance in hallmark pathways among 38 bulk acral melanoma tumors (related to Fig. 2d) and melanoma single-cell transcriptomes from panel a (scRNA-seq), in relation to 22q11.21 amplification status (Methods). NES, normalized enrichment score. **f** Comparison of single-cell differentiation scores determined by CytoTRACE (left) and SCENT (right) in melanoma cells from 22q11.21-amplified (YUJASMIN: $n$ = 312 cells) and non-amplified (YUGRUS: $n$ = 3,786 cells, CytoTRACE $P = 1.21 \times 10^{-22}$, SCENT $P = 5.05 \times 10^{-190}$; YUMASK: $n$ = 15,006 cells, CytoTRACE $P = 4.38 \times 10^{-87}$, SCENT $P = 2.12 \times 10^{-201}$; YUPARK: $n$ = 8,141 cells, CytoTRACE $P = 1.06 \times 10^{-35}$, SCENT $P = 5.18 \times 10^{-198}$) patients using a two-sided, unpaired Wilcoxon rank sum test. **g** Similar to f but shown for the average $\log_2$ expression of pluripotency genes in 22q11.21 amplified and non-amplified acral melanoma cells. In f and g, box center lines, bounds of the box, and whiskers indicate medians, 1st and 3rd quartiles, and minimum and maximum values within 1.5 × IQR (interquartile range) of the box limits, respectively. Statistical significance in f and g was determined by applying a two-sided, unpaired Wilcoxon rank sum test to the data of each non-amplified patient relative to YUJASMIN (YUGRUS $P = 1.36 \times 10^{-21}$, YUMASK $P = 1.27 \times 10^{-22}$, and YUPARK $P = 8.21 \times 10^{-21}$). Source data are provided as a Source Data file.

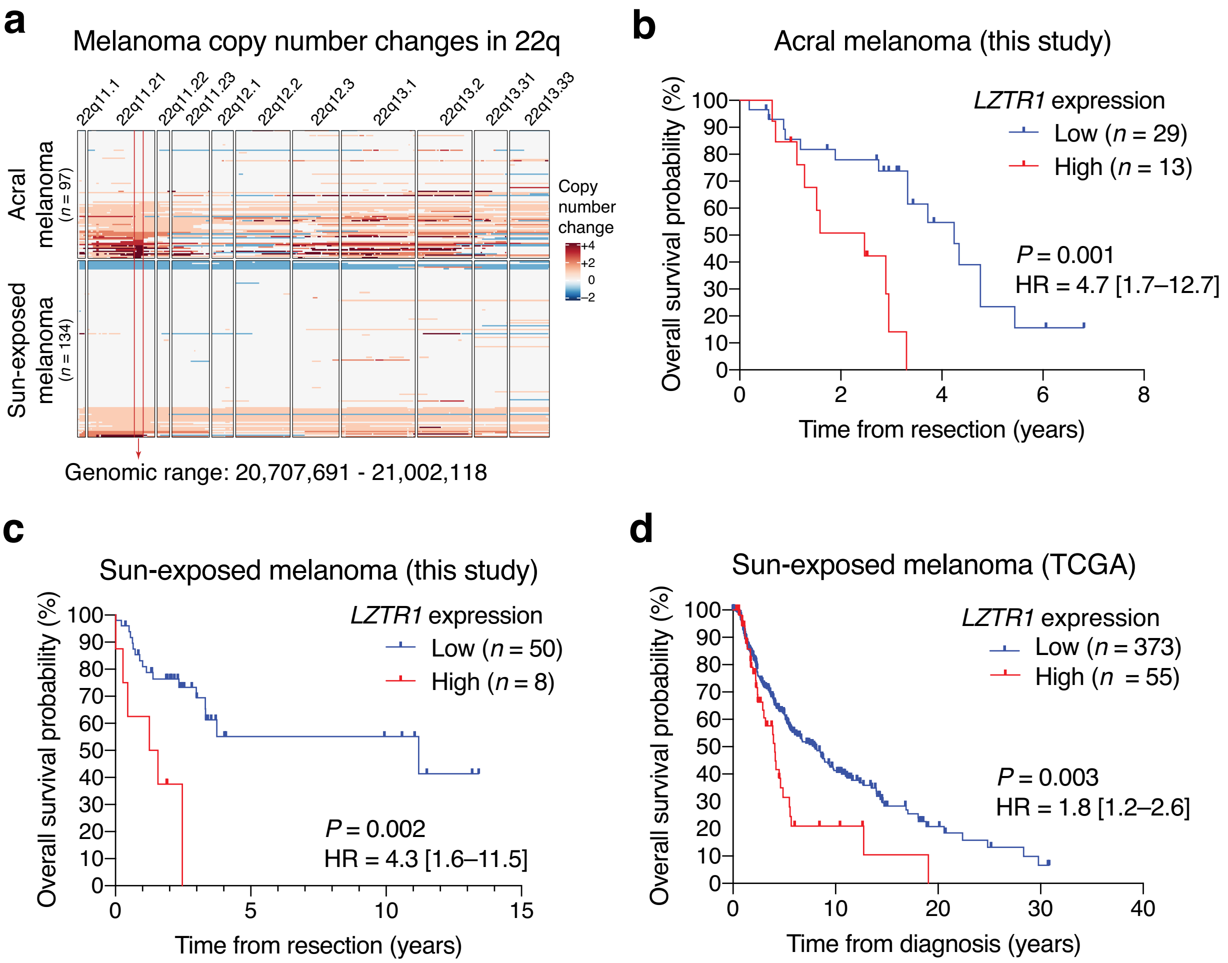


**Supplementary Figure 6: Somatic copy number alterations in 22q and *LZTR1* prognostic associations in melanoma. a** Somatic copy number alterations in the 22q arm of all acral and sun-exposed melanomas profiled by WES in this work. The common region of amplification within cytoband 22q11.21 is indicated by a red box; this region encompasses *LZTR1*. Rows represent melanoma samples and columns indicate genes ordered by location. **b**–**d** Kaplan-Meier plots showing differences in overall survival between LZTR1 high and low patients with acral and sun-exposed melanomas profiled in this work (panels b and c, respectively) and sun-exposed melanomas profiled by TCGA (panel d). LZTR1 was stratified into high and low groups on the basis of copy number status, as described in Methods. Statistical significance was determined by a two-sided log-rank test. HR, hazard ratio. 95% HR is indicated in brackets. Source data are provided as a Source Data file.

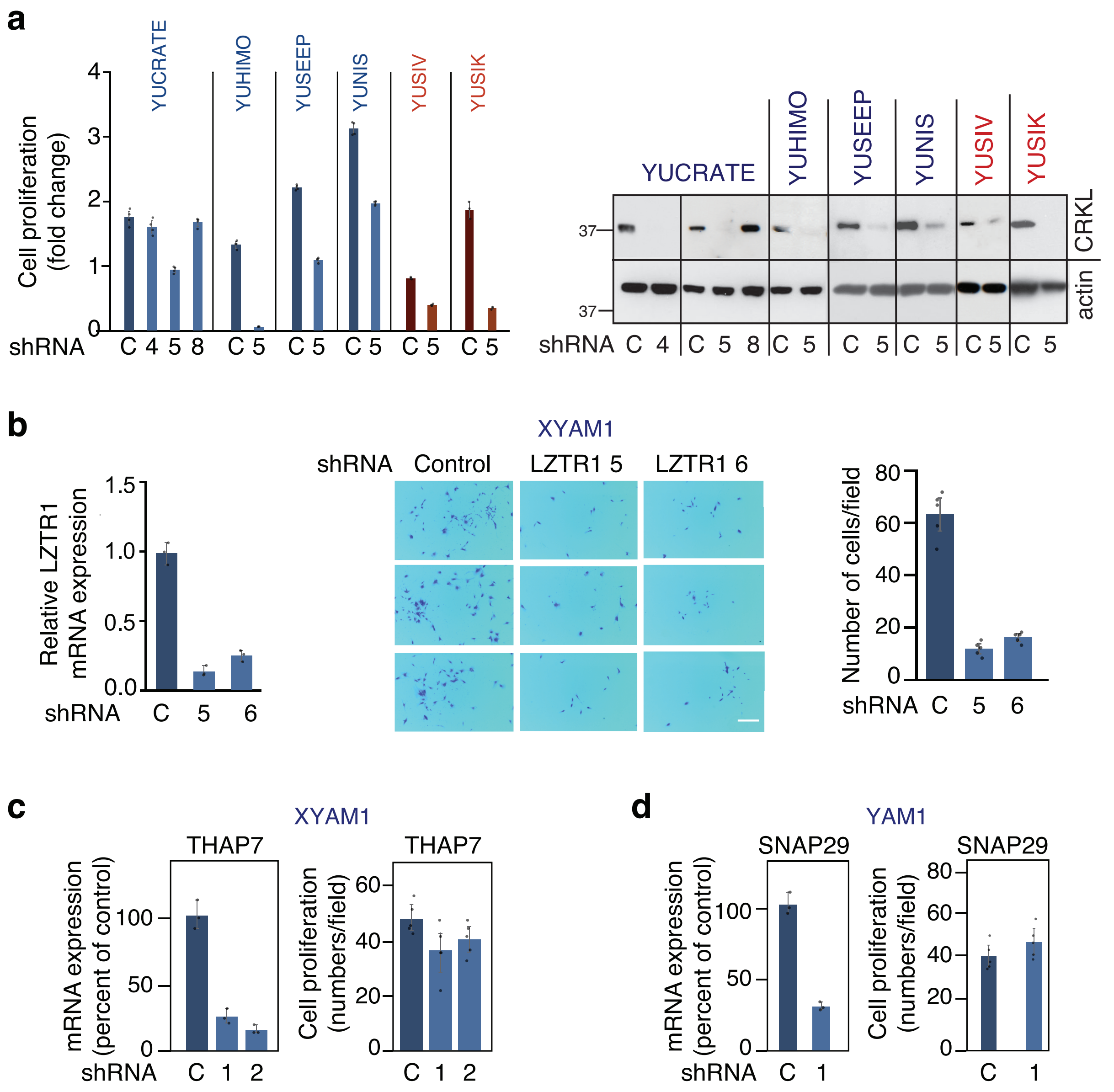


**Supplementary Figure 7: Impact of downregulating chr22q11.21 genes on cell proliferation**. **a** Effect of CRKL shRNAs on cell proliferation (left) and protein expression (right). Cell proliferation in a and b was assessed by a CellTiter-Glo® assay. Bar plots depict fold change between the 3rd and 6th day after infection with CRKL shRNA (numbered), as compared to control (scrambled) shRNA (C). Values are average of *n* = 4 for all cells, except YUSIK (*n* = 3) readings with 95% confidence interval (CI). Data represent two independent experiments. **b** Impact of LZTR1 knockdown on proliferation of XYAM1 acral melanoma cells. Shown are gene expression measured with RT-qPCR (*n* = 3) (left), representative images (middle), and quantification of clonogenic assays (*n* = 5) (right). XYAM1 acral melanoma cells were established from a patient treated in XiangYa Medical School, Changsha, China. Scale bar = 100 μm. Bar Plots on the left and right panels are means of triplicates with 95% CI for LZTR1 shRNA 5 and shRNA 6, respectively. **c**, **d** Knockdown of THAP7 (c) and

SNAP29 (d) had little to no effect on proliferation of XYAM1 acral melanoma cells. Shown are gene expression measured with RT-qPCR (*n* = 3) (left) and quantification of clonogenic assays (*n* = 5) (right). Bar plots show means of triplicates with 95% CI. Cell lines identifiers are indicated above all plots and colored according to their origin: acral melanoma (blue), sun-exposed melanoma (red). Source data are provided as a Source Data file.

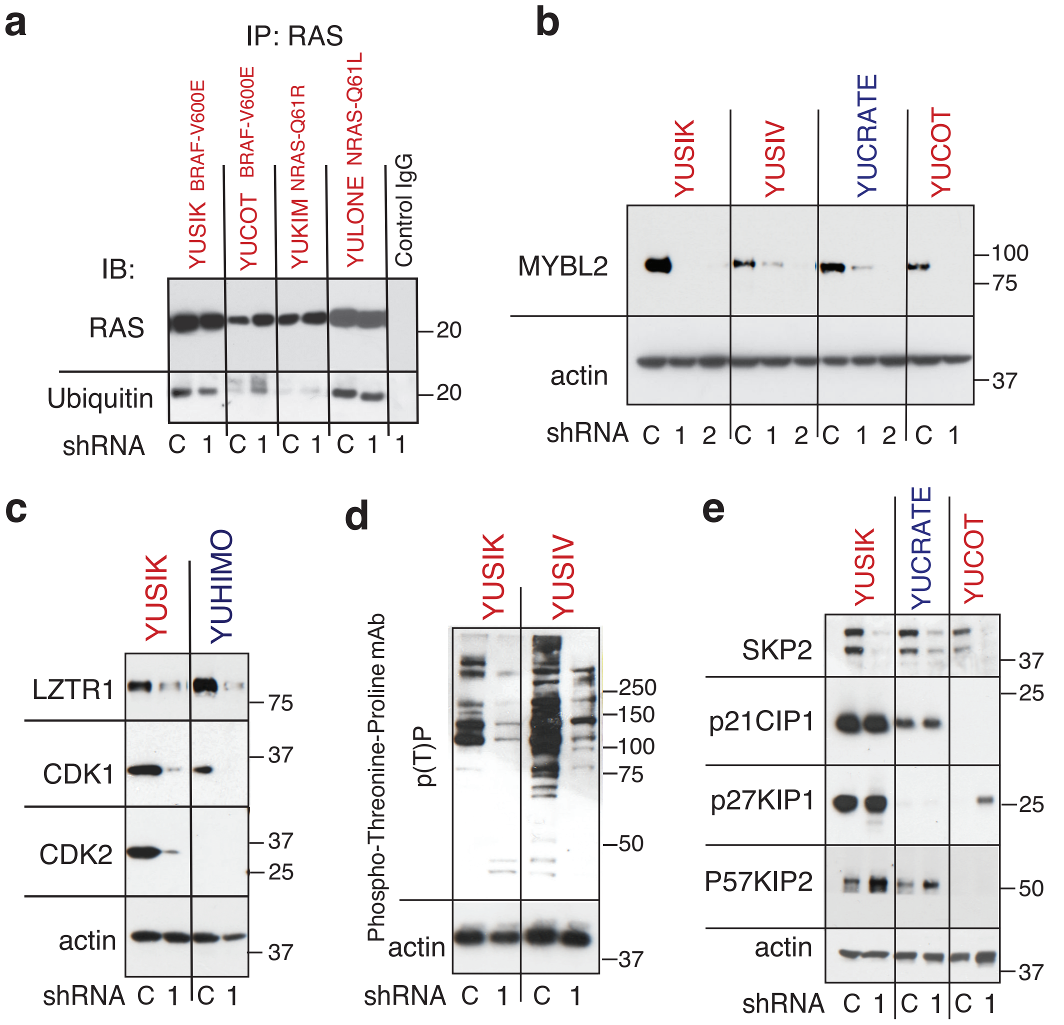


**Supplementary Figure 8: Analysis of cell cycle proteins in response to *shLZTR1*.** **a** RAS ubiquitination is not altered in response to LZTR1 knockdown. Four independent cell lines were treated with shRNA scramble control (C) or *shLZTR1* (1) for 6 days and cell extracts were used to immunoprecipitate RAS (IP) with anti-RAS (mouse monoclonal antibody) and immunoblot with anti-RAS or anti-ubiquitin, rabbit polyclonal antibodies (IB). Immunoprecipitation with IgG using cell extract from YUSIK was used as a control. **b**, **c** Downregulation of cell cycle proteins predicted from GSEA analysis (Fig. 5a). Western blots showing downregulation of MYBL2, CDK1 and CDK2 in response to *shLZTR1*. **d** Loss of LZTR1 suppressed MAPK and CDK families of serine/threonine protein kinases as detected by Phospho-Threonine-Proline (P-Thr-Pro-101) antibodies. **e** Validation of SKP2 downregulation in response to *shLZTR1*. These data exclude major roles for p21CIP1, p27KIP or p57 activation, known to participate in cell arrest, as the basis for LZTR1 growth suppression. Distinct shRNAs are numbered in a-e, as compared to control (scrambled) shRNA (C). Cell line identifiers are indicated above all plots and colored according to their origin: acral melanoma (blue), sun-exposed melanoma (red). Data represent two independent experiments. Source data are provided as a Source Data file.

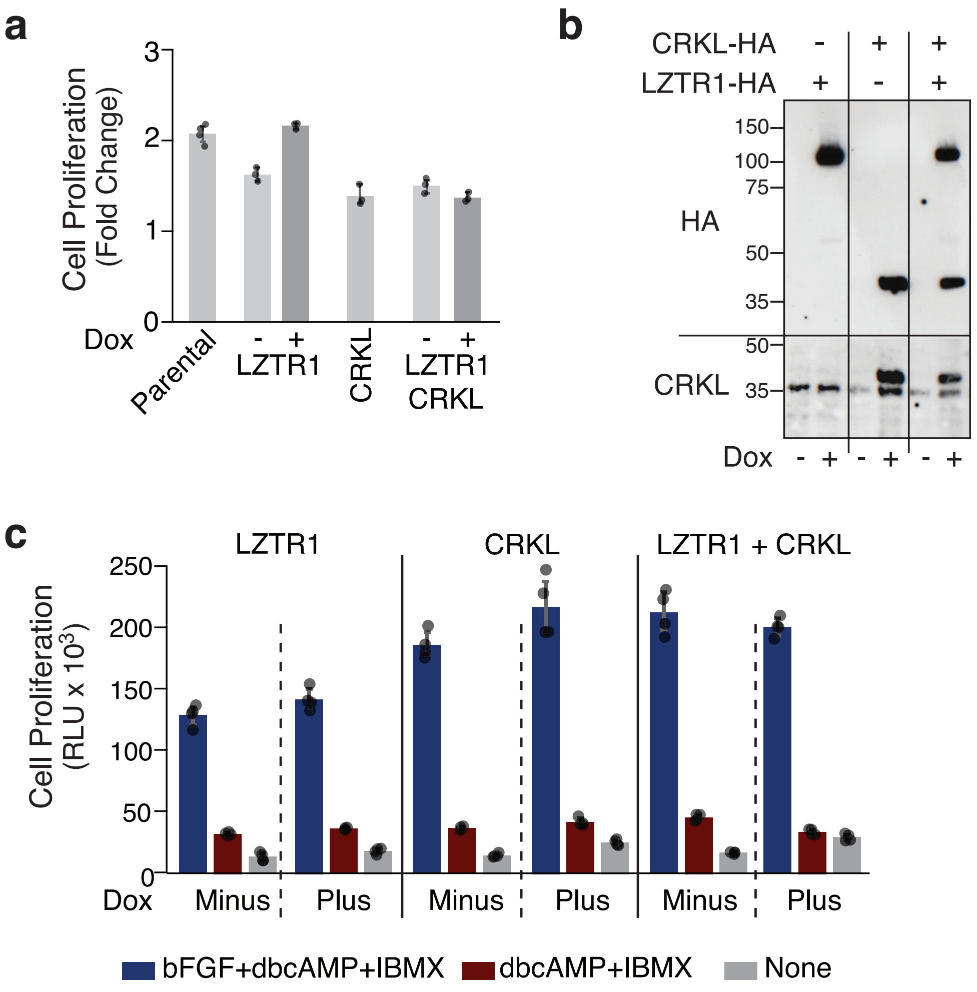


**Supplementary Figure 9: LZTR1 and CRKL, alone and in combination, failed to release human melanocytes from their dependency on growth factors. a** Bar plots representing mean cell viability (CellTiter-Glo® Assay) in response to high expression of LZTR1-HA and PLX304-CRKL in normal human melanocytes (NBMEL C1220), as described in Fig. 6b, with 95% confidence intervals indicated. **b** Western blot showing overexpression of LZTR1-HA and CRKL-HA, alone or in combination, in normal human melanocytes (NBMEL C454). **c** Bar plots showing cell viability (CellTiter-Glo® Assay) performed after three days of incubation in the presence of the three growth factors, bFGF, dbcAMP, and IBMX (blue), the presence of just dbcAMP and IBMX (brown), or in the absence of all three factors (grey). Proliferation was reduced in all conditions relative to bFGF, dbcAMP, and IBMX, and in the absence of growth factors relative to dbcAMP and IBMX. Each measurement is the mean of triplicate (a) or duplicate (c) wells and error bars indicate 95% confidence interval. Where indicated, medium was supplemented with 100 ng/ml doxycycline (Dox). Source data are provided as a Source Data file.

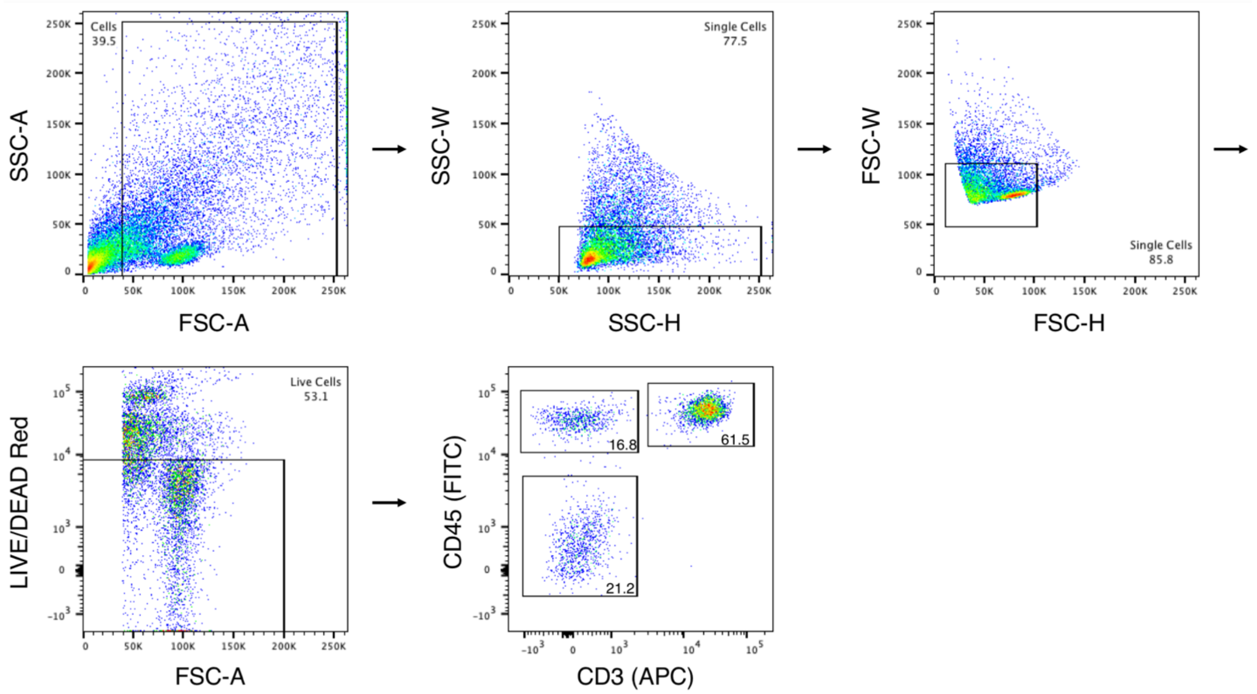


**Supplementary Figure 10: Flow cytometry gating strategy for scRNA-seq profiling of YUJASMIN.** Singlets were identified using FSC (forward scatter) and SSC (side scatter) characteristics as indicated. Viable cells were then gated based on negativity for LIVE/DEAD Red staining. CD3 (APC) and CD45 (FITC) were used to define sorted populations. Sorted $CD3^{+}CD45^{+}$ : $CD3^{-}CD45^{+}$ : $CD3^{-}CD45^{-}$ cells were recombined at a ratio of 2:1:1 for single-cell library preparation.

**Supplementary Table 1. Frequency of SNVs and indels within recurrently mutated genes.**

| Gene | Number of SNVs/indels in Yale cohort (*n* = 32 patients) | Number of SNVs/indels in CSU cohort (*n* = 65 patients) | Total number of SNVs/indels (*n* = 97 patients) | Fisher *P* value (nominal) of difference in SNV/indel prevalence between cohorts |
|---|---|---|---|---|
| *BRAF* | 3 | 5 | 8 | 1.00 |
| *NRAS/HRAS/KRAS* | 8 | 13 | 21 | 0.61 |
| *NF1* | 0 | 3 | 3 | 0.55 |
| *CDKN2A* | 0 | 2 | 2 | 1.00 |
| *KIT* | 5 | 10 | 15 | 1.00 |
| *TP53* | 2 | 2 | 4 | 0.60 |
| *ARID2* | 0 | 4 | 4 | 0.30 |
| *PTEN* | 1 | 1 | 2 | 1.00 |
| *IDH1* | 0 | 0 | 0 | 1.00 |
| *RB1* | 0 | 0 | 0 | 1.00 |
| *RAC1* | 1 | 0 | 1 | 0.33 |
| *MAP2K1* | 1 | 1 | 2 | 1.00 |
| *PPP6C* | 0 | 0 | 0 | 1.00 |
| *DDX3X* | 0 | 1 | 1 | 1.00 |

**Supplementary Table 2. Impact of immune content on the survival association of 22q11.21 amplification**. Prognostic significance of 22q11.21 amplification in acral melanoma, dichotomized as described in Methods, with one of three distinct measures of immune content (CIBERSORTx, Methods) included as a covariate, with or without further adjustment for the indicated stages. Overall survival was calculated as time from resection (Methods) using multivariable Cox proportional hazards regression. TILs, tumor infiltrating lymphocytes; TAMs, tumor associated myeloid cells; Immune, all 22 immune subsets within the LM22 signature matrix used for CIBERSORTx deconvolution (Methods).

| Analysis | Group | Additional covariate | 22q11.21 amplification | | Immune infiltrate | |
|---|---|---|---|---|---|---|
| | | | z score | *p* value | z score | *p* value |
| 22q11.21 Amp + TILs | All stages | None | 2.618 | 0.01 | 0.680 | 0.50 |
| 22q11.21 Amp + TILs | All stages | Stage | 2.661 | 0.01 | 0.779 | 0.44 |
| 22q11.21 Amp + TILs | Stages II, III, IV | None | 2.411 | 0.02 | 0.546 | 0.58 |
| 22q11.21 Amp + TILs | Stages II, III, IV | Stage | 2.503 | 0.01 | 0.680 | 0.50 |
| 22q11.21 Amp + TAMs | All stages | None | 2.564 | 0.01 | -0.483 | 0.63 |
| 22q11.21 Amp + TAMs | All stages | Stage | 2.579 | 0.01 | -0.367 | 0.71 |
| 22q11.21 Amp + TAMs | Stages II, III, IV | None | 2.395 | 0.02 | -0.345 | 0.73 |
| 22q11.21 Amp + TAMs | Stages II, III, IV | Stage | 2.442 | 0.01 | -0.291 | 0.77 |
| 22q11.21 Amp + Immune | All stages | None | 2.512 | 0.01 | -0.536 | 0.59 |
| 22q11.21 Amp + Immune | All stages | Stage | 2.538 | 0.01 | -0.351 | 0.73 |
| 22q11.21 Amp + Immune | Stages II, III, IV | None | 2.364 | 0.02 | -0.388 | 0.70 |
| 22q11.21 Amp + Immune | Stages II, III, IV | Stage | 2.410 | 0.02 | -0.278 | 0.78 |

**Supplementary Table 3. Total copy number alteration burden per sample versus overall survival.** We calculated two metrics capturing total copy number alteration burden (TCNAB): (i) TCNAB v1, the total fraction of the genome contained within CNVs called by GISTIC ($Q < 0.05$); and (ii) TCNAB v2, the sum of all gene-level CNVs (absolute difference from neutral) across the genome. Shown below are univariable Cox proportional hazards regression for TCNAB v1 and v2 alone (top) versus multivariable Cox proportional hazards regression for the covariates indicated in 'Analysis'. Only acral melanomas were analyzed and overall survival was calculated as time from resection (Methods).

| Analysis | Copy number burden | | 22q11.21 amplification | | Stage | |
|---|---|---|---|---|---|---|
| | z score | *p* value | z score | *p* value | z score | *p* value |
| TCNAB v1 (% base pairs altered) | 1.161 | 0.246 | - | - | - | - |
| TCNAB v2 (sum of gene level CNVs) | 2.553 | 0.011 | - | - | - | - |
| TCNAB v1 + 22q11.21 amp + Stage | -1.283 | 0.199 | 2.895 | 0.004 | 3.611 | 3.05E-04 |
| TCNAB v2 + 22q11.21 amp + Stage | 0.559 | 0.576 | 2.372 | 0.018 | 3.038 | 0.002 |

**Supplementary Table 4. Impact of distinct CNV callers on the prognostic significance of 22q11.21 amplification.** *Top:* Univariable prognostic significance of 22q11.21 amplification in acral melanoma, dichotomized as described in Methods, for CNVs identified by three approaches (CNVL [Methods], GISTIC, ABSOLUTE). Overall survival was calculated as time from resection using Cox proportional hazards regression (Methods). *Bottom*: Same as above but for multivariable Cox models combining 22q11.21 amplification and TIL content as covariates, with and without adjustment for the indicated stages. ABS, ABSOLUTE; TILs, tumor infiltrating lymphocytes.

| Analysis | Group | Additional covariate | 22q11.21 amplification | | TILs | |
|---|---|---|---|---|---|---|
| | | | z score | *p* value | z score | *p* value |
| CNVL 22q11.21 Amp | All stages | None | 2.881 | 0.004 | - | - |
| GISTIC 22q11.21 Amp | All stages | None | 2.645 | 0.008 | - | - |
| ABS 22q11.21 Amp | All stages | None | 2.089 | 0.037 | - | - |
| | | | | | | |
| CNVL 22q11.21 Amp + TILs | All stages | None | 2.618 | 0.009 | 0.680 | 0.496 |
| CNVL 22q11.21 Amp + TILs | All stages | Stage | 2.661 | 0.008 | 0.779 | 0.436 |
| CNVL 22q11.21 Amp + TILs | Stages II, III, IV | None | 2.411 | 0.016 | 0.546 | 0.585 |
| CNVL 22q11.21 Amp + TILs | Stages II, III, IV | Stage | 2.503 | 0.012 | 0.680 | 0.496 |
| GISTIC 22q11.21 Amp + TILs | All stages | None | 2.405 | 0.016 | 0.579 | 0.563 |
| GISTIC 22q11.21 Amp + TILs | All stages | Stage | 2.366 | 0.018 | 0.629 | 0.529 |
| GISTIC 22q11.21 Amp + TILs | Stages II, III, IV | None | 2.190 | 0.029 | 0.439 | 0.661 |
| GISTIC 22q11.21 Amp + TILs | Stages II, III, IV | Stage | 2.215 | 0.027 | 0.516 | 0.606 |
| ABS 22q11.21 Amp + TILs | All stages | None | 2.677 | 0.007 | 1.040 | 0.298 |
| ABS 22q11.21 Amp + TILs | All stages | Stage | 2.650 | 0.008 | 1.144 | 0.253 |
| ABS 22q11.21 Amp + TILs | Stages II, III, IV | None | 2.452 | 0.014 | 0.875 | 0.381 |
| ABS 22q11.21 Amp + TILs | Stages II, III, IV | Stage | 2.470 | 0.014 | 0.973 | 0.331 |

**Supplementary Table 5. scRNA-seq quality control parameters.**

| Sample | Min nCount | Max nCount | Maximum percent reads mapped to mitochondrial genes | Total cells after quality control |
|---|---|---|---|---|
| YUJASMIN | 500 | 25000 | 5 | 7059 |
| YUPARK | 500 | 20000 | 15 | 8509 |
| YUMASK | 500 | 15000 | 10 | 15454 |
| YUGRUS | 500 | 10000 | 15 | 3831 |

**Supplementary Table 6. Correlation between expression and copy number for genes located within the chr22q11.21 focal amplification wide peak in acral melanomas.** See also Fig. 2h.

| | Wilcoxon test statistic | Wilcoxon test P-value | Spearman correlation coefficient | Spearman correlation P-value |
|---|---|---|---|---|
| *LZTR1* | 301.00 | 0.0001009 | 0.73 | 2.48727E-07 |
| *SNAP29* | 288.00 | 0.0009662 | 0.66 | 5.64777E-06 |
| *KLHL22* | 282.00 | 0.0012724 | 0.65 | 1.2308E-05 |
| *ZNF74* | 282.00 | 0.0012724 | 0.64 | 1.31921E-05 |
| *P2RX6* | 278.00 | 0.0020149 | 0.59 | 8.26527E-05 |
| *PI4KA* | 270.00 | 0.0021285 | 0.59 | 8.22789E-05 |
| *COMT* | 280.00 | 0.0023189 | 0.54 | 0.000412719 |
| *RANBP1* | 274.00 | 0.0056293 | 0.55 | 0.000334449 |
| *SERPIND1* | 269.50 | 0.005964 | 0.49 | 0.001601616 |
| *TRMT2A* | 273.00 | 0.006204 | 0.60 | 6.26152E-05 |
| *C22orf29* | 270.00 | 0.0064885 | 0.57 | 0.00018125 |
| *TXNRD2* | 266.00 | 0.0094657 | 0.56 | 0.00021996 |
| *DGCR8* | 267.00 | 0.0108515 | 0.56 | 0.000232833 |
| *CRKL* | 262.00 | 0.0114308 | 0.55 | 0.000363424 |
| *ZDHHC8* | 256.00 | 0.0225149 | 0.52 | 0.000730217 |
| *THAP7* | 249.50 | 0.0308961 | 0.50 | 0.001506323 |
| *DGCR6L* | 247.00 | 0.03601 | 0.40 | 0.013549536 |
| *SCARF2* | 245.00 | 0.04184 | 0.44 | 0.005956955 |
| *MED15* | 244.00 | 0.0450343 | 0.45 | 0.004819058 |
| *ARVCF* | 246.00 | 0.0548138 | 0.45 | 0.004382698 |
| *AIFM3* | 233.00 | 0.0951252 | 0.43 | 0.007674917 |
| *GNB1L* | 211.00 | 0.3517065 | 0.29 | 0.072642574 |
| *RTN4R* | 199.50 | 0.4945082 | 0.14 | 0.394688126 |
| *RIMBP3* | 184.00 | 0.8243254 | 0.09 | 0.576336421 |

**Supplementary Table 7. Characteristics of melanoma cell lines.** *Melanoma exome sequencing data are available via dbGaP (phs000933.v2.p1) https://www.ncbi.nlm.nih.gov/projects/gap/cgi-bin/study.cgi?study_id=phs000933.v2.p1

| Cell Line | Tumor Type | Mutation BRAF-NRAS | Other Key Mutations | References* |
|---|---|---|---|---|
| YUCRATE | Acral | BRAF p.G469A | MAPK6 p.E520K | 2 |
| YUHIMO | Acral | PDE4DIP-BRAF fusion | CDKN2A loss; CDKN2B loss; TERT Gain; LZTR1 Gain; CRKL Gain, EP300 Gain; SOX10 Gain; SRC Gain | 2 |
| YUSEEP | Acral | GOLGA4-RAF1 fusion | EWSR1 p.G290E; CDKN2A loss; CDKN2B loss; TERT Gain; CCND1 Gain; LZTR1 Gain; CRKL Gain, EP300 Gain; SOX10 Gain | This work |
| XYAM1 | Acral | WT | TP53 duplication | Acral melanoma cells isolated from a patient treated in XiangYa Hospital, Changsha, China. This work |
| YUSIK | Sun-exposed | BRAF p. V600E | MAP2K3 p.P162L; PTEN p.E288K | 2 |
| YUKIM | Sun-exposed | NRAS p. Q61R | CTNNB1 p.S33F; CDK11 p.E477V; BRCA2 p.R2799K; PTEN p.E288K; SMARCA4 p.R1135Q/p.A1423V; INSR V1047I | 2 |
| YUGASP | Sun-exposed | NRAS p.Q61L | NF1 LOH; CDKN2A-loss; FANCA p.P667L; MITF null | 1 |
| YUSIV | Sun-exposed | PDE8A-RAF1 fusion | NF1 p.L626F; BRCA1 p.V772A; PTEN p.E288K; TRRAP p.S722F; PCDHGA1 p.P155L | 1 |
| YUCOT | Sun-exposed | BRAF p. V600E | NF1 W784X-L-W, SPRED1 Q365X, MAPK6- E407X and K408R, PIK3C2B R1349Q, MC1R V60L a R163Q (1) D294A | 2 |
| YURKEN | Sun-exposed | BRAF p. V600E | NF1 P228S, MAP3K11- R118W; CDKN2A-loss, MC1R V60L-heterozygous, MC1R R151C-heterozygous | 2 |
| SK-MEL-28 | Sun-exposed | BRAF p. V600E | SK-MEL-28: Human Melanoma Cell Line (ATCC HTB-72) | 3 |
| YULONE | Sun-exposed | NRAS p.Q61L | MAP2K3- P267S, ARID1A P1135L; ARID3B R402C, TRRAP L2257F, FBXW7 R465C, MC1R V60L-homozygous | This work |
| NBMEL (226, 454, 1220) | Normal melanocytes | WT | None | This work |
| C57BL | Immortalized mouse melanocytes isolated from the epidermis of newborn C57BL mice | NA | NA | 4 |

## Supplementary References


1. Krauthammer, M., *et al*. (2015) Exome sequencing identifies recurrent mutations in NF1 and RASopathy genes in sun-exposed melanomas. *Nat Genet* **47**, 996-1002.
2. Krauthammer, *et al*. (2012) Exome sequencing identifies recurrent somatic RAC1 mutations in melanoma. *Nat Genet* **44**, 1006-1014.
3. ATCC https://www.atcc.org/~/media/PDFs/Culture%20Guides/Cell_Lines_by_Gene_Mutation.ashx
4. Kwon, B. S., *et al*. (1995) Mouse silver mutation is caused by a single base insertion in the putative cytoplasmic domain of Pmel 17. *Nucleic Acids Res* 23, 154-158.
