# Supplementary material for: Integrative molecular and clinical profiling of acral melanoma links focal amplification of 22q11.21 to metastasis
Source: Nat Commun. 2022 Feb 23;13:898. doi: 10.1038/s41467-022-28566-4 (PMC8866401; doi:10.1038/s41467-022-28566-4)
Supplement: Supplementary file 2 — Description of Additional Supplementary Files [file 41467_2022_28566_MOESM2_ESM.pdf]

Description of Additional Supplementary Files

File Name: Supplementary Data 1
Description: **Patient demographics and clinical/sequencing characteristics.** a Patient demographics and clinical characteristics. b Characteristics of whole-exome sequencing data. c Characteristics of bulk RNA sequencing data.

File Name: Supplementary Data 2
Description: **Frequencies of mutations in patients and subtypes.** a Frequency of SNVs and indels per patient. b MutSigCV results for acral melanoma. c MutSigCV results for sun-exposed melanoma.

File Name: Supplementary Data 3
Description: **Mutation, fusion, copy number and COSMIC data.**

File Name: Supplementary Data 4
Description: **Copy number amplification analysis.** a Catalog of focal amplifications identified in acral and sun-exposed melanoma by GISTIC 2.0 ($Q<0.05$). b Recurrence frequencies of focal amplifications. To calculate recurrence frequencies, we calculated the average gene-level copy number (Copy Number Matrix in Supplementary Data 3) per GISTIC wide peak. We subtracted 2 from all gene-level values so that copy numberneutral regions were equal to 0. We retained peaks with frequency ≥5% in either subtype. c Random forest analysis of focal amplifications in acral versus sun-exposed melanoma samples. The 40 most informative cytobands as determined by a random forest (RF) model. Here a RF with 500 trees was applied to distinguish acral from sun-exposed melanoma samples using gene-level copy-number data binarized at copy number ≥4. Gene-level feature importance was assessed by mean decrease in accuracy. Cytoband ranking was assigned based on the maximum mean decrease in accuracy achieved by any gene contained within the respective cytoband, and the number of genes in each cytoband is indicated in the parentheses.

File Name: Supplementary Data 5
Description: **Copy number deletion analysis**. a Catalog of focal deletions identified in acral and sun-exposed melanoma by GISTIC 2.0 ($Q<0.05$). b Recurrence frequencies of focal deletions. To calculate recurrence frequencies, we calculated the average gene-level copy number (Copy Number Matrix in Supplementary Data 3) per GISTIC wide peak. We subtracted 2 from all gene-level values so that copy numberneutral regions were equal to 0. We retained peaks with frequency ≥5% in either subtype.

File Name: Supplementary Data 6
Description: **Frequency of fusion genes by melanoma subtype.**

File Name: Supplementary Data 7
Description: **Survival associations of somatic alterations, related to Figure 2a.** Z-score calculations and nomenclature for GISTIC peak IDs are described in Methods.

File Name: Supplementary Data 8
Description: **Lentiviral vectors MISSION pLKO.1 puromycin bearing shRNA and CRISPR sgRNA used to test the effect of gene-specific downregulation on cell proliferation.**

File Name: Supplementary Data 9
Description: **Antibodies**
