# Supplementary material for: Integrative molecular and clinical profiling of acral melanoma links focal amplification of 22q11.21 to metastasis
Source: Nat Commun. 2022 Feb 23;13:898. doi: 10.1038/s41467-022-28566-4 (PMC8866401; doi:10.1038/s41467-022-28566-4)
Supplement: Supplementary file 12 — Reporting Summary [file 41467_2022_28566_MOESM12_ESM.pdf]

nature research

Corresponding author(s): Ruth Halaban, Aaron M. Newman, Xiang Chen

Last updated by author(s): Jan 25, 2022

Reporting Summary

Nature Research wishes to improve the reproducibility of the work that we publish. This form provides structure for consistency and transparency in reporting. For further information on Nature Research policies, see our Editorial Policies and the Editorial Policy Checklist.

Statistics

For all statistical analyses, confirm that the following items are present in the figure legend, table legend, main text, or Methods section.

n/a	Confirmed	
☐	☒	The exact sample size (*n*) for each experimental group/condition, given as a discrete number and unit of measurement
☐	☒	A statement on whether measurements were taken from distinct samples or whether the same sample was measured repeatedly
☐	☒	The statistical test(s) used AND whether they are one- or two-sided<br>*Only common tests should be described solely by name; describe more complex techniques in the Methods section.*
☐	☒	A description of all covariates tested
☐	☒	A description of any assumptions or corrections, such as tests of normality and adjustment for multiple comparisons
☐	☒	A full description of the statistical parameters including central tendency (e.g. means) or other basic estimates (e.g. regression coefficient) AND variation (e.g. standard deviation) or associated estimates of uncertainty (e.g. confidence intervals)
☐	☒	For null hypothesis testing, the test statistic (e.g. *F*, *t*, *r*) with confidence intervals, effect sizes, degrees of freedom and *P* value noted<br>*Give P values as exact values whenever suitable.*
☒	☐	For Bayesian analysis, information on the choice of priors and Markov chain Monte Carlo settings
☒	☐	For hierarchical and complex designs, identification of the appropriate level for tests and full reporting of outcomes
☐	☒	Estimates of effect sizes (e.g. Cohen's *d*, Pearson's *r*), indicating how they were calculated

Our web collection on statistics for biologists contains articles on many of the points above.

Software and code

Policy information about availability of computer code

Data collection	No specific software was used for data collection beyond software associated with commercially available hardware (e.g., Agilent Bioanalyzer, Illumina sequencing instruments, etc.), including BD FACS Diva v8.0.1.
Data analysis	Software packages used in this study are detailed in Methods, including Cell Ranger v3.0.2 or 5.0.1, GISTIC v2.0.23, TrimGalore v0.3.7, FastQC v0.11.2, BWA MEM v0.7.15, Picard v2.17.11, Annovar, VEP v95, HISAT2 v2.1.0, STAR-Fusion v1.2.0, FusionInspector v1.1.0, vcf2maf v1.6.14, Salmon v0.99, GATK v3.2, MutSigCV v1.41, ABSOLUTE v1.0.6, inferCNV v1.6.0, CIBERSORTx v1.0.41, CytoTRACE v0.3.3, SCENT v1.0.2, and various R v3.5+ packages (e.g., tximport v1.10.1, sva v3.30.1, Seurat v4.0.4, survminer 0.4.5, biomaRt v2.40.5, ComplexHeatmap v1.20.0, survival v2.44-1.1+), Python v3.8.5, and Graphpad Prism v7 or v8. Custom scripts used in this study are publicly available from https://github.com/knightjimr/CNVL.

For manuscripts utilizing custom algorithms or software that are central to the research but not yet described in published literature, software must be made available to editors and reviewers. We strongly encourage code deposition in a community repository (e.g. GitHub). See the Nature Research guidelines for submitting code & software for further information.

Data

Policy information about availability of data

All manuscripts must include a data availability statement. This statement should provide the following information, where applicable:

- Accession codes, unique identifiers, or web links for publicly available datasets
- A list of figures that have associated raw data
- A description of any restrictions on data availability

The Liang et al. publicly available WES data used in this study are available in the dbGAP database under accession code phs001036.v1.p1 https://www.ncbi.nlm.nih.gov/projects/gap/cgi-bin/study.cgi?study_id=phs001036.v1.p1. All raw DNA sequencing data and raw bulk/single-cell RNA sequencing data

generated in this study from the Yale cohort have been deposited in the dbGAP database under accession code phs000933.v3.p1 https://www.ncbi.nlm.nih.gov/projects/gap/cgi-bin/study.cgi?study_id=phs000933.v3.p1. The data are available under restricted access, access can be obtained by submitting a project request to dbGAP (https://dbgap.ncbi.nlm.nih.gov/aa/wga.cgi). All raw DNA and RNA sequencing data generated in this study from the CSU cohort have been deposited in the Genome Sequence Archive (GSA) database under accession code HRA001648 https://bigd.big.ac.cn/gsa-human/browse/HRA001648. The data are available under restricted access, access can be obtained by submitting a request to the corresponding Data Access Committee (Contact person: Peng Cong, Email: pengcongxy@csu.edu.cn). The processed bulk RNA sequencing data (Yale and CSU cohorts) and processed single-cell RNA sequencing data (Yale cohort) have been deposited in the Gene Expression Omnibus (GEO) database under accession code GSE162682 https://www.ncbi.nlm.nih.gov/geo/query/acc.cgi?acc=GSE162682. The remaining data are available within the Article, Supplementary Information, or Supplementary Data files. Source data are provided with this paper.

Field-specific reporting

Please select the one below that is the best fit for your research. If you are not sure, read the appropriate sections before making your selection.

- [x] Life sciences
- [ ] Behavioural & social sciences
- [ ] Ecological, evolutionary & environmental sciences

For a reference copy of the document with all sections, see nature.com/documents/nr-reporting-summary-flat.pdf

Life sciences study design

All studies must disclose on these points even when the disclosure is negative.

Sample size

No sample-size estimates were performed to ensure adequate power to detect a pre-specified effect size. Patient cohort sizes were based on the availability of tumor specimens within a retrospective analysis. Nevertheless, our conclusions are supported by a wide variety of technical and clinically-relevant data analyses, several of which contained a large number of patient samples and all of which were analyzed and interpreted using statistically appropriate techniques, as described in Methods.

Data exclusions

For DNA and RNA sequencing, samples with a yield of ≥0.5 ng/µl were used for sequencing. This threshold was pre-established. All analyzed genomic profiles generated in this work satisfied quality control thresholds described in Methods.

Replication

We analyzed newly generated genomic data from two acral melanoma cohorts, one from Yale and one from CSU. The finding that 22q11.21 is adversely prognostic in acral melanoma was consistent across both cohorts. Additionally the finding that 22q11.21 amplification frequency is linked to lymph node involvement remained consistent across the Yale and CSU cohorts as well as a third cohort of acral melanoma tumors profiled by an independent study (Liang et al.).

Randomization

Sample groups were determined according to the experimental question and known or predetermined biological or clinical phenotypes. No randomization was applied as our cohorts were retrospective. Nevertheless, we adjusted for stage and immune content as potential confounding variables when calculating the prognostic significance of 22q11.21 focal amplification (Supplementary Tables 2 and 4).

Blinding

The investigators were not blinded to group (e.g., patient cohort) allocation during data collection or analysis, however all cohorts analyzed in this work were independently generated without prior knowledge of patient outcomes or genomic features, including 22q11.21 amplification status and LZTR1 expression levels.

Reporting for specific materials, systems and methods

We require information from authors about some types of materials, experimental systems and methods used in many studies. Here, indicate whether each material, system or method listed is relevant to your study. If you are not sure if a list item applies to your research, read the appropriate section before selecting a response.

Materials & experimental systems

n/a	Involved in the study	
[ ]	[x]	Antibodies
[ ]	[x]	Eukaryotic cell lines
[x]	[ ]	Palaeontology and archaeology
[x]	[ ]	Animals and other organisms
[ ]	[x]	Human research participants
[x]	[ ]	Clinical data
[x]	[ ]	Dual use research of concern

Methods

n/a	Involved in the study	
[x]	[ ]	ChIP-seq
[ ]	[x]	Flow cytometry
[x]	[ ]	MRI-based neuroimaging

Antibodies

Antibodies used

Antibodies, including supplier name, clone name, dilutions used, and catalog number, are provided in Supplementary Data 9. The following antibody dilutions were used: Beta-Actin (Mouse mAb, AC-15), 1/5000; RAS (Rabbit mAb, EP1125Y), 1/20; LZTR1 (Mouse mAb E-12), 1/200; LZTR1 (Rabbit pAb), 1/1000; CRKL (Mouse mAb, 32H4), 1/1000; HA-tag (Mouse mAb, 6E2), 1/1000; RAS, RAS10 (Mouse mAb), 1/500; BRAF (Goat IgG), 1 µg/mL; phospho-B-Raf (Ser445), 1/1000; phospho-p44/42 MAPK (Erk1/2, Thr202/Tyr204), 1/1000; p44/42K MAPK (Erk1/2) (Mouse mAb, 3A7), 1/1000; p44/42K MAPK/Erk1/2 (Rabbit pAb), 1/1000; MEK1/2 (Mouse mAb, L38C12), 1/1000; Phospho-MEK1/2 (S217/221) (Rabbit pAb), 1/1000; p38 MAPK kinase (Rabbit mAb, D13E1), 1/1000; phospho-

	p38MAPK (T180/Y182) (Rabbit pAb), 1/1000; PARP1 (Rabbit mAb, 46D11), 1/1000; IKKβ (NFKB1) (Rabbit mAb, D30C6), 1/1000; p27KIP1 (Mouse mAb), 1/2000; p21 Waf1/Cip1 (Rabbit mAb, R.229.6), 1/1000; RBL2 (Rabbit mAb, D9T7M), 1/1000; RB (Mouse mAb, IF8), 1/200; CDC2 (Rabbit pAb), 1/1000; CDK2 (Rabbit mAb, 78B2), 1/1000; Phospho-Threonine-Proline (Mouse mAb, P-Thr-Pro-101), 1/1000; P57 KIP2 (Rabbit pAb), 1/1000; Ubiquitin (Rabbit pAb), 1/1000; p53 (Mouse mAb, D0-7), 1/500; GOLGA4/GCP2 (Rabbit pAb), 1/1000; MITF (Mouse mAb clone 5), 1/500; GM130 (Mouse mAb, 4A3), 1/100; B-Myb (Mouse mAb, LX015.1), 1 μg/mL; Skp2 (Mouse mAb), 2 μg/mL; Tyrosinase (Goat pAb (M-19), 1/200, Calnexin (Mouse mAb), 1/50; Actin (rhodamine-phalloidin), 1/1000; SRC (Mouse mAb, 5D10C4), 1/500; Phospho-Src Family (Tyr416) (Rabbit pAb), 1/1000; E-cadherin (Polyclonal Goat IgG), 0.5 μg/mL; N-Cadherin (Mouse mAb, 13A9), 1/1000; Integrin β1 (Rabbit mAb D6S1W), 1/1000: CD45 (Mouse IgG1, clone H130), 1/100; APC anti-human CD3 (Mouse IgG1, clone HIT3a), 5/100.
Validation	Antibodies were purchased from certified commercial sources, each of which validated antibody quality by probing extracts from different cell types showing the correct size protein band. We also validated the antibodies by employing several methods. For example, we validated the anti-LZTR1 (E-12, catalog no. sc-390166, Santa Cruz Biotechnology, Inc.) by Western blot analysis with and without blocking with LZTR1 (D-1) peptide SC-390731P. In addition, we confirmed the antibodies by knocking down gene expression with several specific shRNAs (represented in Fig. 3 for LZTR1 and Supplementary Fig. 7a for CRKL). Finally, we confirmed the antibodies by immunoblotting exogenously expressed LZTR1-HA, or CRKL-V5, as described in Fig. 6b. Anti-RAS (EP1125Y, Rabbit pAb, catalog no. ab52939, ABCAM Cambridge, UK) was validated in our hands by immunoprecipitation and immunoblotting with two different antibodies: Anti-Ras (EP1125Y) ABCAM cat #ab52939 and LSBio C99434 anti NRAS antibodies. Anti-GM130 antibody (clone 4A3, MilliporeSigma, St. Louis, MO) and Anti-Calnexin (mouse mAb, StressGen Biotechnologies Corporation, Victoria, British Columbia, Canada) were validated by the respective manufacturers and by us. We used two different antibodies to validated GM130: GM130 (D6B1) XP® Rabbit mAb #12480 CST and mouse mAb Anti-GM130 clone 4A3 from Millipore. Antibodies were applied at 1:200 to 1:2,000 dilutions, as recommended by the manufacturer. Validation of Alexa Fluor® 488 anti-human CD45 antibody (clone H130, catalog no. 304019, BioLegend) and APC anti-human CD3 antibody (clone HIT3a, catalog no. 300319, BioLegend) was performed by the manufacturer using immunocytochemistry analyses and by our group, as described below in "Flow Cytometry".

Eukaryotic cell lines

Policy information about cell lines

Cell line source(s)	SK-MEL-28 (HTB-72™) and 293T (HEK-293) were purchased from the American Type Culture Collection (ATCC, Manassas, VA). All other melanoma cell lines, including normal human and mouse melanocytes, were acquired from the Specimen Resource Core of Yale SPORE in Skin Cancer. This core facility collects tissues with informed consent for research use. The Yale Department of Pathology confirms the origin of the tumors. The core tests for the presence of any contaminants, such as fibroblasts, bacteria, and mycoplasma. Contaminated cells are discarded and the equipment, hoods, and incubators undergo thorough disinfection.
Authentication	Melanoma cell lines were authenticated by whole exome sequencing (Supplementary Table 7) or bulk RNA sequencing (Fig. 5a), as appropriate. 293T cells made lentiviruses, as expected. SK-MEL-28 was validated by confirmation of a BRAF V600E mutation, MITF and KIT expression, and pigmentation.
Mycoplasma contamination	All cell lines tested negative for mycoplasma contamination (see above).
Commonly misidentified lines (See ICLAC register)	No cell lines used in this study are commonly misidentified.

Human research participants

Policy information about studies involving human research participants

Population characteristics	Human subjects are described in Methods and in Supplementary Data 1a.
Recruitment	No patients were specifically recruited for this study. All patient specimens were previously banked and retrospectively analyzed in an agnostic manner without any pre-specified hypothesis, mitigating the risk of bias, including self-selection bias.
Ethics oversight	All patient samples were collected with informed consent for research use and were approved by the Yale University and Central South University Institutional Review Boards in accordance with the Declaration of Helsinki.

Note that full information on the approval of the study protocol must also be provided in the manuscript.

Flow Cytometry

Plots

Confirm that:

- [x] The axis labels state the marker and fluorochrome used (e.g. CD4-FITC).
- [x] The axis scales are clearly visible. Include numbers along axes only for bottom left plot of group (a 'group' is an analysis of identical markers).
- [x] All plots are contour plots with outliers or pseudocolor plots.
- [x] A numerical value for number of cells or percentage (with statistics) is provided.

Methodology

Sample preparation	A freshly resected surgical tumor sample (patient YUJASMIN) was dissociated and sorted in the following ratios prior to 10x Chromium library preparation: 50% CD3+CD45+ T cells: 25% CD3–CD45+ non-T immune cells: 25% CD45– stromal/cancer cells. Cell viability was assessed by the LIVE/DEAD™ Fixable Red Dead Cell Stain Kit (catalog no. L34971, Thermo Fisher). The following antibodies were used: Alexa Fluor® 488 anti-human CD45 antibody (clone H130, catalog no. 304019, BioLegend); APC anti-human CD3 antibody (clone HIT3a, catalog no. 300319, BioLegend).
Instrument	BD FACSAria Flow Cytometer
Software	Flow cytometry data were collected with BD FACSDIVA software version 8.0.1 from BD Biosciences.
Cell population abundance	Purities were assessed by scRNA-seq and found to positively correlate with expected proportions (above).
Gating strategy	Live/dead discrimination was performed using the LIVE/DEAD™ Fixable Red Dead Cell Stain Kit (catalog no. L34971, Thermo Fisher). Cell subsets were gated as described above. The gating strategy is shown in Supplementary Figure 10.

☒ Tick this box to confirm that a figure exemplifying the gating strategy is provided in the Supplementary Information.
